# Natural Hydrophobic Deep Eutectic Solvent-Based Enhanced Extraction of Bioactive Compounds from *Cannabis sativa* L. Leaf for Pharmaceutical Applications

**DOI:** 10.3390/ijms27072933

**Published:** 2026-03-24

**Authors:** Serwat Naz, Sumia Akram, Rabia Naeem, Haroon Iftikhar, Rizwan Ashraf, Noor Ul Ain Khalid, Muhammad Shahid, Imad A. Abu-Yousef, Amin F. Majdalawieh, Muhammad Mushtaq

**Affiliations:** 1 Department of Chemistry, Government College University Lahore, Lahore 54000, Pakistan; serwat432@gmail.com (S.N.); rabianaeem@gcu.edu.pk (R.N.); chemistharoon@yahoo.com (H.I.); chemistnoor@yahoo.com (N.U.A.K.); 2 Division of Science and Technology, University of Education Lahore, Lahore 54770, Pakistan; sumia.akram@ue.edu.pk; 3 Department of Chemistry, University of Agriculture, Faisalabad 38000, Pakistan; rizi_chem82@hotmail.com; 4 Department of Biochemistry, University of Agriculture, Faisalabad 38000, Pakistan; mshahiduaf@yahoo.com; 5 Department of Biology, Chemistry and Environmental Sciences, College of Arts and Sciences, American University of Sharjah, Sharjah P.O. Box 26666, United Arab Emirates; amajdalawieh@aus.edu; 6 Advanced Biosciences and Bioengineering Research Center, American University of Sharjah, Sharjah P.O. Box 26666, United Arab Emirates; 7 Bioinformatics and Computational Biology Research Group, American University of Sharjah, Sharjah P.O. Box 26666, United Arab Emirates

**Keywords:** hydrophobic deep eutectic solvent, sustainable and efficient extraction, antioxidant, antidiabetic, biofilm inhibition, cytotoxicity

## Abstract

*Cannabis sativa* L. leaves (CSL) are a rich in bioactive compounds and known for their medicinal and recreational uses. In this study, a natural hydrophobic deep eutectic solvent (HDES) system composed of menthol and thymol (1:1) was employed for the efficient extraction of bioactive compounds from CSL. Extraction of bioactives was optimized at various conditions involving DES/ethanol ratio, temperature, and extraction time, as well as shaking speed through statistical models including response surface methodology (RSM) and artificial neural network (ANN). The maximum bioactive yield, equal to 70% (*w*/*w*) of powdered CSL, was achieved at optimized values of 5.5 mL DES, 4.5 mL ethanol, and 225 rpm shaking speed at 55 °C for 107.5 min. It was observed that slightly adjusting the shaking speed and temperatures customized the nature of bioactives with more antioxidant, antidiabetic, and antimicrobial properties. The extracts of CSL produced while applying natural HDES were found to be non-toxic during hemolytic assay. Overall, HDES when mixed with ethanol in 55:45 ratio produced CSL extracts with an ample level of phenolics (133.75 mg GAE/g) and flavonoids (120.05 mg QE/g). GC-MS analysis of CSL extracts produced by HDES revealed the presence of multiple bioactives like tetrahydrocannabivarin, cannabidiol, cannabinol, cannabidivarol, dl-menthol, levomenthol, and 4-hydroxy-3-methylacetophenone. Based on these findings, it can be concluded that HDES in combination with ethanol may work as an efficient extraction solvent to recover CSL bioactives without compromising their antioxidant features and safety for use in food and pharmaceutical applications.

## 1. Introduction

*Cannabis sativa* L. (*C. sativa*) is an aromatic wild plant that grows all around the world, famous for its recreational and medicinal use and its distinct aroma and flavor [1]. According to Carl Linnaeus’ nomenclature, *C. sativa* falls among the most abundant and famous cultivars of cannabis grown for fiber, hemp oil, fragrance and medicinal uses [2]. *C. sativa* leaves may contain more than 500 potential bioactives of flavonoid, cannabinoids, and terpenoids families [3]. The ratio of Δ9-tetrahydrocannabinol (THC) to cannabidiol (CBD) is followed for the chemotaxonomy of the plant [4]. *C. sativa* contains Δ9-THC, as the predominant cannabinoid, compared to other members of this family rich in CBD cannabinoid, which predominantly discriminates this between marijuana and hemp. Many important phytochemical classes such as cannabinoids, terpenes and flavonoids possessing medicinal traits are reported in the leaf of *Cannabis sativa* L. (CSL) [5].

Antioxidants, especially of phenolics, flavonoids and cannabinoids, are reported to be effective in controlling oxidative stress-related disorders. These antioxidants can neutralize free radicals, produced either through stress or environmental factors like pollution and UV radiation, and reduce the risk of cell damage, chronic illness, and neurological problems [6]. Diabetic Mellitus type II is a dysregulation of glucose often caused by increased levels of stress hormones like cortisol and epinephrine or cell damage in the pancreas that decreases the production of insulin. According to a published report of the World Health Organization (WHO), over 422 million people are currently suffering from diabetes [7]. Diabetes is linked with insulin resistance or insufficient production of insulin in the body, which can increase the blood glucose level and lead to kidney failure, cardiac arrest and nerve damage. Likewise, obesity has been linked with oxidative stress in the body caused by the overproduction of free radicals, either due to stress or environmental factors. WHO statistics reveal a three folds increase in obesity since 1975, with more than 650 million adults in the obese class [8].

Deep Eutectic Solvent (DES) is an emerging class of non-volatile, tunable, benign, and eco-friendly solvents [9]. DES can be made up of hydrogen bond donors (HBD) and hydrogen bond acceptors (HBA), whose ratio customizes physicochemical attributes like the density, viscosity, polarizability and surface tension of the final mixture [10]. DES is replacing organic solvents and ionic liquids, due to its tailorable features and simple preparation methods [11]. Researchers are nowadays trying to fortify food and pharmaceutical products with natural antioxidants to enhance their shelf life and health benefits. This strategy demands more intense sources of natural antioxidants and/or an efficient and sustainable technique that can extract antioxidants without deteriorating their inherent antioxidant potential. The conventional extraction techniques involving the use of organic solvents have been ruled out either due to health issues or environmental safety concerns. Solid phase extraction and many modern extraction techniques, like supercritical fluid extraction, enzyme-assisted extraction, and membrane-based extractions, face economic and scalability issues [12,13].

Various research groups have already reported deep eutectic solvent-based systems for the extraction of phenolic compounds from olive mill wastewater [14], carotenoids from tomato [15], etc. However, the presence of moisture in these extracting systems makes the handling of extracts difficult or, in adverse cases, deteriorates the antioxidant quality during storage [16]. This entire scenario calls for the development of alternative extracting materials. Therefore, the present research uses a natural hydrophobic deep eutectic solvent (HDES) made up of menthol and thymol as an extraction solvent for the recovery of bioactive antioxidants from *C. sativa* leaf (CSL). The extraction process was exhaustively investigated over a wide range of extraction conditions to modulate and optimize the bioactive recovery. To assess the effectiveness of selected HDES as extraction solvents, CSL extracts were subsequently evaluated for their *in*-*vitro* antioxidant, antidiabetic, and antimicrobial activities and hemolytic activity. Finally, gas chromatography coupled with mass spectrometry (GC-MS) was applied to establish the presence of bioactives. The outcomes of the present study may provide a cascade of opportunities regarding the extraction of pharmaceutically active bioactive compounds from plant and herbal resources.

## 2. Results and Discussion

### 2.1. FTIR Analysis

**Figure 1:**
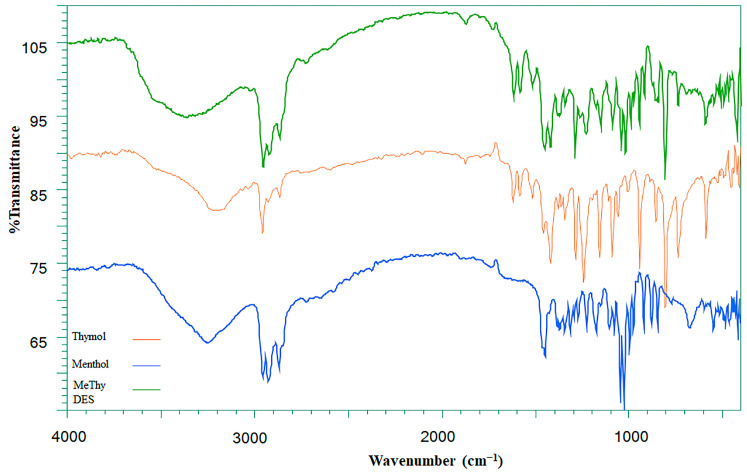
The comparison of FTIR spectra of HDES with its precursors differentiated with different colors (thymol: orange, menthol: blue, DES: green).

The successful synthesis of the HDES solvent system was confirmed through FTIR spectroscopy, which indicated the formation of menthol–thymol-based HDES as obvious by the appearance of prominent peaks associated with hydrogen bond formation. A peculiar peak around 3232 cm^−1^ in the HDES mixture (Figure 1) indicates strong O–H hydrogen bonding. The change in the intensity of this peak can be attributed to the change in the strength of hydrogen bond between menthol and thymol. The presence of peaks at 2958, 2924, 2870, and 2846 cm^−1^ (Figure 1) represents the aliphatic C–H stretching vibrations, reflecting the methyl and methylene groups that are present in menthol and thymol. Additional peaks at 1462 and 1446 cm^−1^ further confirm the presence of C–C stretching within the benzene ring. The region between 1300 and 1100 cm^−1^ exhibits characteristic stretching vibrations associated with C–C and C–O bonds as well as isopropyl groups, common to both precursors. No new functional groups were observed in the DES spectrum, which indicates that its formation is only due to physical interactions (hydrogen bonding).

### 2.2. Extraction of CSL Bioactives

The extraction of CSL bioactives through HDES extraction medium was conducted over a wide range of extraction conditions to optimize the extract yield, which is affected by various factors including the shaking speed (A), DES ratio (B), temperature (C), and extraction time (D). When the CSL bioactives obtained under various conditions were subjected to analysis of variance (ANOVA), the outcomes assembled in Table 1 indicate the linear effects of these parameters (A, B, C, and D), their first order interaction (AB, AC, AD, BC, and BD), and quadratic effects (A^2^, B^2^, C^2^, and D^2^) significantly (*p* ≤ 0.05) affected the extraction of CSL bioactives. Therefore, these parameters were modulated to form a quadratic equation to calculate the bioactive yield as

$$\begin{array}{c} Extract\;Yield=+1.20++0.17A++0.22B-0.08C-0.21D-0.21AB+0.14AC\\ \;-0.23AD+0.22BD+0.05CD-0.18A^{2}-0.17B^{2}-0.08C^{2}-0.0827D^{2} \end{array}$$

**Figure 2:**
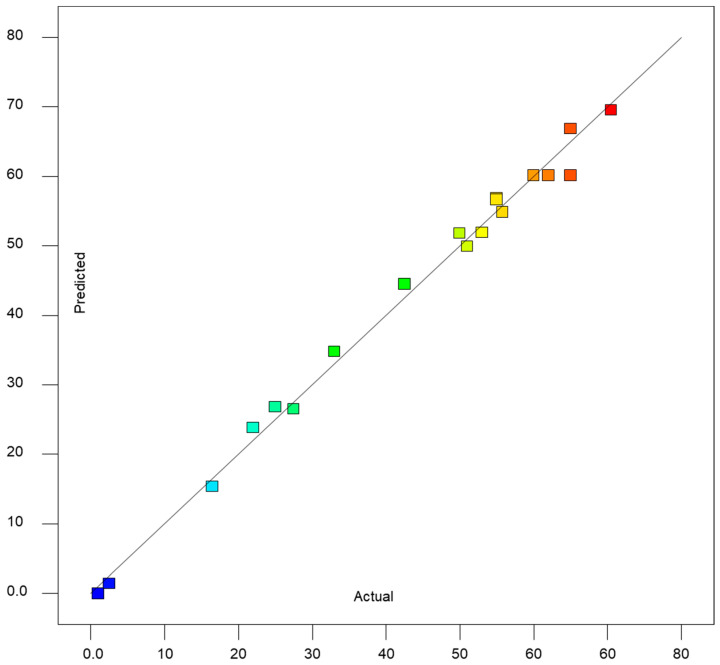
A comparison of CSL bioactive yield (g/100 g of CSL powder) observed during actual experiments and those predicted by the regression equation above.

The data plotted in Figure 2 indicates there was an excellent agreement (R^2^ ≥ 0.099) between the yield predicted by this equation and that observed during the actual experiments.

**Figure 3:**
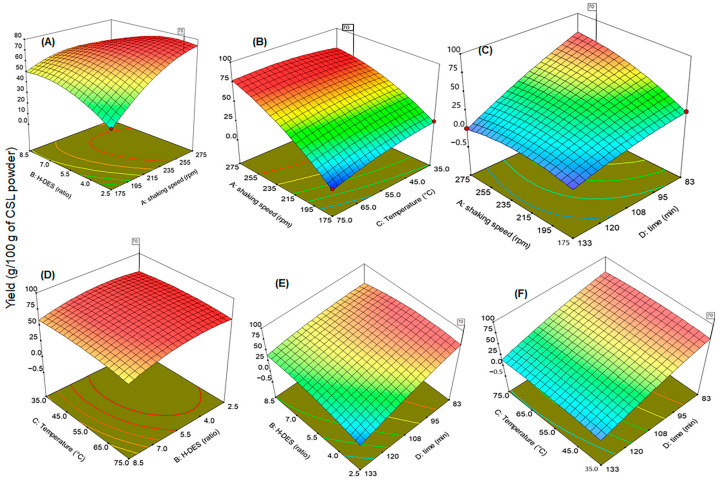
Three-dimensional response surface graphs to elaborate the synergism between tested extraction conditions of shaking speed (**A**); RMP, HDES (**B**); RMP, temperature (**C**); RMP, extraction time (**D**); HDES, temperature (**E**); HDES, time (**F**); temperature, time, respectively applied to optimize the extraction of CSL bioactive.

The effect of these parameters on the extraction yield was studied in different sets of paired parameters, and the results are described as three-dimensional response surface plots, as given in Figure 3A–F. These paired interactions illustrate the interactive effects on the percentage yield of CSL bioactives. The interactive effect of shaking speed and HDES ratio is presented in Figure 3A, where an increase in shaking speed and a decrease in the HDES ratio significantly (*p* < 0.05) improved the extraction yield that enhanced up to 70 g/100 g of CSL powder at optimal values of shaking speed 202.5 rpm and an HDES ratio of 2.5. Similarly, when the interactive influence of shaking speed and temperature were studied, higher shaking speeds and lower temperatures of extraction medium offered higher extraction efficiency, as given in Figure 3B. The data presented in Figure 3C describe the interaction between the shaking speed and extraction time. The paired interaction indicates that extraction at a reduced extraction time and increased shaking speed offered higher recovery rates. Figure 3D illustrates the direct relationship between the DES ratio and temperature of the medium, which was linear with temperature up to 45 °C, after which it declined. The 3D response built in Figure 3E shows the interaction between the DES ratio and extraction time, indicating that decreasing the extraction time while maintaining a lower DES ratio resulted in higher yields. Finally, Figure 3F shows that the combined effect of increasing the extraction time at a high temperature of the medium can reduce the overall yield. This adverse effect could be associated with the degradation of phytochemicals at a high temperature [17].

The negative quadratic coefficients in the equation suggest that exceeding optimal levels results in decreased efficiency. However, this equation provided limited output about the relative significance of individual factors.

The experimental results showed that increasing the shaking speed enhances the efficiency of extraction solvent, while a higher DES-to-ethanol ratio reduces the extraction yield. Among the studied parameters, the optimal yield was achieved at a shaking speed of 202.5 rpm with a DES/ethanol ratio of 2.5. The significance of the model was estimated through analysis of variance (ANOVA) which confirmed the adequacy of the model with an F-value of 59.89 and a very low probability (*p* < 0.0001) as given in Table 1. Other interactions of the studied parameters, including linear effects (A, B, C, D), interactions (AB, AC, AD, BD, CD), and quadratic terms (A^2^, B^2^, C^2^, D^2^), were also found to be significant with *p*-values ≥ 0.05, indicating a strong effect on extraction yield (described in Figure 3). The lack of fit of test model was not found to be significant (F = 4.50; *p* ≥ 0.0946), suggesting the best fit of the model for the given data. Overall, the model was declared as robust statistically and suitable for optimizing the extraction parameters.

**Figure 4:**
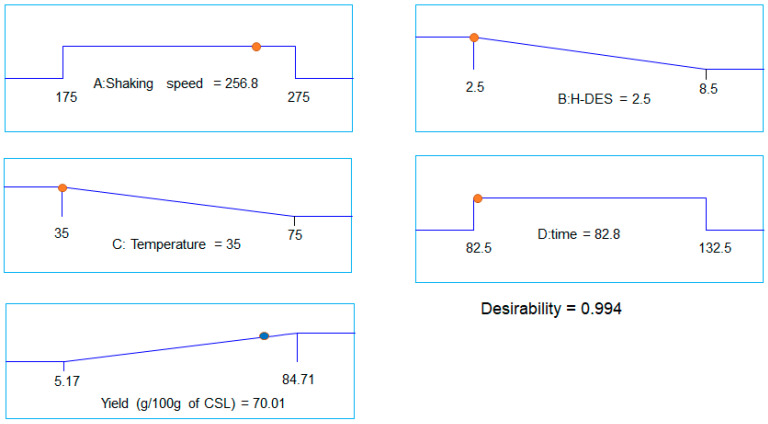
The desirability ramp of best solution (desirability ≥ 90%) offered by response surface optimization of HDES-based extraction of CSL.

The overall analysis of the experimental data revealed that increasing the shaking speed, with an optimum HDES ratio, extraction time, and moderate temperature, resulted in a higher extraction yield and enriched bioactive of CSL. The maximum yield 70 g/100 g of CSL powder was observed under the optimized conditions of 202.5 rpm shaking speed, a DES ratio of 2.5, an extraction time of 82.5 min, and a temperature of medium of 35 °C, as shown in Figure 4.

### 2.3. Comparison of RSM and ANN

The RSM results were used in ANN for further analysis. All ANN-predicted results are given in Table 2.

**Figure 5:**
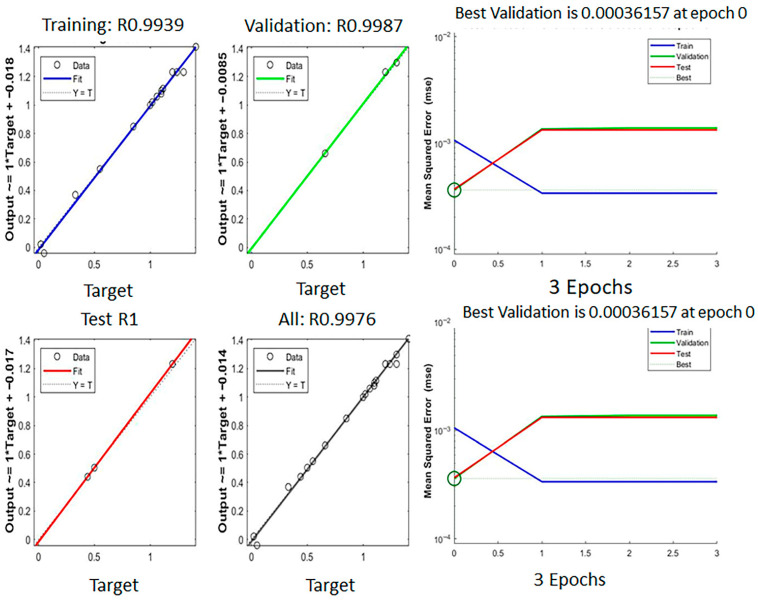
The performance, regression, and training state by ANN modeling for % yield from *Cannabis sativa* L. leaves.

The ANN showed excellent predictive performance by achieving training, testing and validation R-values higher than 0.99, and the All-R value is 0.99761 (illustrated in Figure 5). At epoch 0, the best validation performance (MSE) of 0.00036157, was observed and the maximum gradient descent observed was 2.885 × 10^−10^ at epoch 3. These results ensure the robustness of the ANN model in predicting the extraction yield.

### 2.4. Comparison of Modeling

The comparison of the two statistical methods as predictive tools was conducted using various parameters, including R^2^, %AAD, and RMSE. For carbon capturing, the R^2^ value obtained by RSM is 0.9925, while for ANN it is also 0.9952. The %AAD value for RSM is 9.85 and 2.30 for ANN. The RMSE obtained for RSM is 0.035, while for ANN, it is 0.029. The percentage prediction error (PPE) of RSM is 8.50, while for ANN, it is 4.99. All these values are given in Table 3.

The predictions made by both RSM and ANN were in an acceptable range of PPE, indicating that these methods aligned well with the experimental results. Table 3 and Table 4 show the relationship between the experimental value obtained by RSM and its comparison with the RSM and ANN predicted values. The optimal conditions for finding higher yield are 5.5 mL DES and 4.5 mL ethanol at 55 °C, with a shaking speed of 225 rpm for 107.5 min.

Based on the RSM and ANN comparison, it can be concluded that both models effectively predicted the yield values, aligning closely with the experimental results. A slightly better result and closer predictions are shown by ANN, with a high R^2^ (0.9952), lower AAD (2.30%), PPE (4.99%), and RMSE (0.029) compared to RSM. Although both models showed reliable results, ANN’s lower error values and higher prediction accuracy highlight its strength in handling complex and nonlinear relationships. Moreover, the ANN model appears to be a more reliable tool for optimization of yield due to its adaptability, accuracy and superior error minimization making it ideal for data-driven optimization tasks.

### 2.5. TPC and TFC Results

Table 5 presents the results regarding the total phenolic contents (TPC) and total flavonoid contents (TFC) levels found in the CSL extracts obtained while applying HDES made up of menthol and thymol (1:1) as the extraction solvents. It is obvious from the assembled data that the CSL extracts produced while applying HDES as solvent contained higher levels of TPC 133.07 ± 1.87 mg GAE/g of CSL extract as compared to ethanol and water, which furnished CSL extracts containing 9.52 ± 0.23 and 3.26 ± 1.02 mg/GAE TPC, respectively. Hydrophobic deep eutectic solvent (HDES) has not been previously utilized as an extraction solvent to recover CSL bioactives. However, the TPC in the CSL extracts produced by ethanol and water (Table 5) were comparable with previous reports by Drinić et al. [18] and Ahmed et al. [19], who have applied water and organic solvent to extract the phenolic bioactive form of CSL. Overall, it was observed that the HDES-based extraction solvent offers good solubilities for phenolic bioactives; however, its higher viscosity obstructs the mass transfers, which can be overcome by applying higher shaking speeds or mixing DES with lighter (low density) solvents like ethanol.

As with TPC, the extracts of CSL produced while applying HDES + ethanol as an extraction solvent contained significantly (*p* ≤ 0.05) higher levels of TFC 108.05 ± 8.89 mg QuE/g of CSl extract. The experimental findings assembled in Table 5 described a decrease in bioactives with the addition of ethanol in the extraction solvent or an alteration in the shaking speed, probably associated with compromised mass transfer rates. The maximum levels of TPC and TFC in CSL extracts were found when they were extracted applying solvent mixture of 5.5 mL HDES and 4.5 mL ethanol at 55 °C, with a shaking speed of 225 rpm for 107.5 min. No research work has been reported regarding DES-based extraction CSL phenolics; however, the levels of TPC and TFC obtained while applying water and organic solvents-based extraction techniques [18,19] were comparable with those observed for water as an extraction solvent in the present study.

### 2.6. RSC of CSL Extracts

**Figure 6:**
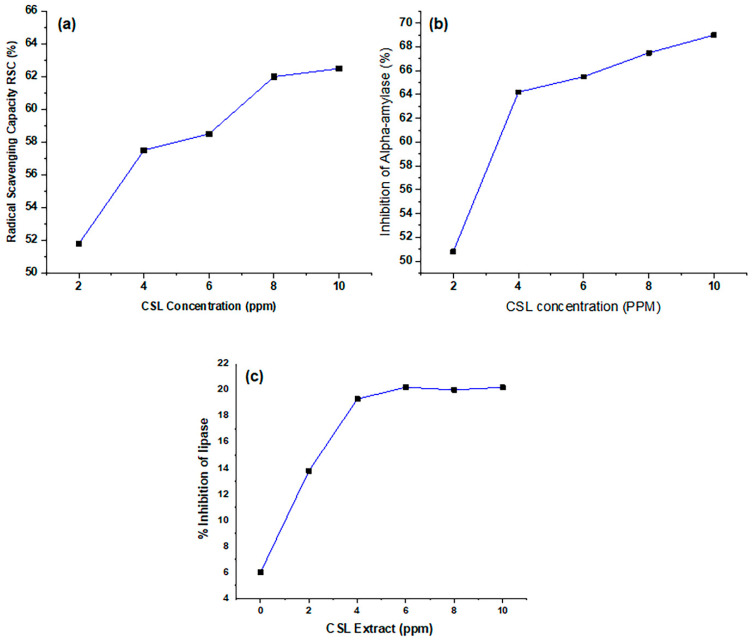
Radical scavenging capacity (**a**), inhibition of α-amylase activity (**b**) and lipase inhibition potential (**c**) of CSL extracts produced by HDES.

The graph in Figure 6a illustrates the radical scavenging capacity (RSC) of *C. sativa* leaf (CSL) extracts obtained by applying HDES. A clear positive correlation was observed between the concentration of the CSL extract and its RSC, indicating a dose-dependent relationship between the concentration and the inhibition of free radicals. Specifically, with the increase in CSL concentration, the RSC increases exponentially until the CSL concentration reaches 2 ppm, which is an RSC equal to 52%. The RSC increased linearly with a further increase in CSL concentration, with a maximum inhibition equal to 64.5% offered by the 10 ppm solution of CSL extracts obtained via HDES. This trend can be better understood from the data plotted in Figure 6a. It should be mentioned here that no work has been undertaken to explore the effectiveness of HDES for the extraction of bioactives from CSL. In a previous study, Cásedas et al. [20] have noted that ethanolic and aqueous extracts of CSL can inhibit 100% of DPPH free radicals at 60 and 97 PPM, respectively. The elevated percentage inhibition of DPPH free radicals offered by a smaller concentration of CSL extracts obtained by hydrophobic DES indicates their superior antioxidant quality. Unlike the study cited above, we have observed a maximum of 64.5% inhibition of DPPH free radicals; the 100% inhibition of DPPH free radicals might be due to prolonged incubation, which leads to the neutralization of free radicals.

### 2.7. Inhibition of α-Amylase

The inhibition potential of the CSL extracts was investigated against α-amylase to explore the bioactivity of the HDES-based extracts. The graph in Figure 6b demonstrates the relationship between the concentration (ppm) and alpha-amylase inhibitory activity, which exhibits a higher increase in the percentage of inhibition with the increase in concentration, followed by less steep regions. This kind of trend indicates that either the extracts are rich in antioxidants or the α-amylase inhibition potential of the CSL bioactives produced via HDES-based extraction is higher as compared to those obtained using conventional organic solvents. With the increase in the CSL extracts’ concentration from 2 to 4 ppm, a sheer increase in % inhibition of α-amylase was noted, but a further increase in the CSL extract level could not cause the inhibition of α-amylase at parallel rates. Similar kinds of behavior have also been noted for the DPPH radical scavenging behavior of CSL extracts produced via hydrophobic DES extraction. Another aspect of this infers the cleaner nature of the CSL extracts produced via HDES-based extraction. It was observed that the % inhibition of α-amylase increased in a dose-dependent way with a higher step rate for smaller concentrations of CSL extracts and vice versa. A keen review of the literature published regarding CSL phytochemistry and biological activities indicates no study has been undertaken for the DES-based extraction of CSL bioactives and their antihyperglycemic activities. Haddou et al. [21] tested CSL extracts prepared in ethanol, *n*-hexane, dichloromethane, and ethyl acetate for their inhibitory effect on alpha-amylase. The % inhibition of α-amylase offered by these CSL extracts when applied below 200 PPM was lower than that of the HDES-based CSL extract at 2.0 PPM. The higher % inhibition of α-amylase offered by the smaller concentration of HDES-based CSL extracts indicates their cleaner nature as compared to those produced by conventional solvent extraction.

### 2.8. Lipase Inhibition Activity Results

Figure 6c shows the in vitro lipase inhibitory effects of CSL extracts produced while applying hydrophobic deep eutectic solvent (HDES) as an extraction solvent. As with the RSC and inhibition of α-amylase, the % lipase inhibition increases with the increase in CSL extract concentration. The data show a higher rate of change for smaller extract concentrations. At 0 ppm (control), the baseline inhibition was approximately 6%, which increased to around 14% at 2 ppm and further to 19.5% at 4 ppm, reflecting the contributing role of CSL extracts imparting lipase inhibition activity. The inhibition reached a peak of approximately 20.3% at 6 ppm, after which it showed minimal variation, with values of 19.7% at 8 ppm and 20.2% at 10 ppm. These results interestingly confirm the dose-dependent increase in lipase inhibitory activity followed by a saturation effect at higher concentrations. These results overall confirm that the sample exhibits inhibitory potential, with maximum effectiveness observed between 6 and 10 ppm. No D-DES-based extraction of CSL bioactive has been reported for comparison; however, Haddou et al. [21] used CSL extracts prepared in ethanol, *n*-hexane, dichloromethane, and ethyl acetate to inhibit the lipase enzyme, and their observed values were exceptionally high, with inhibition equal to 59, 61, and 75% for *n*-hexane-, ethyl acetate-, and dichloromethane-based extracts of CSL, which might due to a prolonged incubation time.

### 2.9. Hemolytic Activity Results

The hemolytic activity of HDES-based CSL extracts was measured to investigate the toxicity level against erythrocytes and expressed as the percentage hemolysis in comparison to the positive control (Triton-X). All tested samples demonstrated low to moderate hemolytic effects, indicating minimal toxicity. Among the extracts, the control sample showed the lowest hemolytic activity (5.02%), whereas the CS DES extract obtained using DES showed a slightly higher activity (8.85%). Triton X-100 was used as a positive control and demonstrated a high hemolytic activity of 83.3%, confirming the assay’s sensitivity. No significant toxic effects of CSL extracts have been previously reported.

### 2.10. Biofilm Inhibition Results

The results revealed that the ethanolic extract of CSL exhibited the highest biofilm inhibition activity at 47.8%, whereas the HDES-based extract showed inhibition at 39%. Despite this, the DES extract demonstrated a notable anti-biofilm effect against *Staphylococcus epidermidis* and inhibited biofilm formation by *Staphylococcus aureus* ATCC 35556; however, the antibacterial activity appeared to be only partially related to membrane disruption. Additionally, *CS* extracts were found to influence both pathogenic bacteria (Gram-positive and Gram-negative) and beneficial probiotic strains such as *Lactobacillus* and *Bifidobacterium*.

### 2.11. Phytochemical Profile of CSL Extracts 

In the present study, gas chromatography–mass spectrometry (GC-MS) was employed to identify the phytochemical constituents of CSL extracts obtained via HDES-based extraction. Although GC-MS is limited to detecting the volatile compounds listed in its library database, it remains a reliable and practical technique, especially for identifying compounds present in low concentrations that are difficult to isolate for techniques like NMR or high-resolution MS. The GC-MS analysis of the HDES–ethanol extract of CSL revealed several key bioactive compounds (Table 6), including flavonoids, terpenoids, and cannabinoids identified through NIST library as given in Table S2. Among the identified compounds, the significant amount (46.48% relative area) of 4-hydroxy-3-methylacetophenone was identified as the major component, showing a retention time (RT) of 12.468 min in the chromatogram. Terpenoids were also observed in significant amounts such as dl-menthol (19.93%) and levomenthol (9.67%) detected at 8.362 and 8.503 min of RT, respectively. Cannabinoids like cannabidiol, cannabichromene, cannabidiol, ∆^9^-tetrahydrocannabivarin, cannabispirin, ∆^8^-tetrahydrocannabinol, and cannabinol are also present in the DES-extract of CS leaves, as described in Table 6. These findings align with previous reports that highlight a diverse cannabinoid and terpene profile in CSL confirming its phytochemical richness and therapeutic potential.

## 3. Materials and Methods

### 3.1. Chemicals and Reagents

All chemicals used in this study were of analytical grade and sourced exclusively from Sigma-Aldrich (St Louis, MO, USA) and Merck (Darmstadt, Germany). The sodium dihydrogen phosphate (NaH_2_PO_4_ ≥ 99%), sodium chloride (NaCl ≥ 99.5%, Merck), dimethyl sulfoxide (DMSO ≥ 99.9%), 3,5-dinitrosalicylic acid (DNS ≥ 99%), α-amylase (from *Aspergillus oryzae*, ≥30 units/mg), menthol (≥99.9%), thymol (≥99.6%), Tris buffer (≥99.8%), olive oil (analytical grade), orlistat (≥98%), gallic acid (≥98%), sodium carbonate (Na_2_CO_3_ ≥99.5%), para-nitrophenyl phosphate (≥98.4%) and Folin–Ciocalteu (10 M) reagent were supplied by local vendors. Additionally, terephthalic acid (≥98%), absolute ethanol (≥99.8%), quercetin hydrate (≥95%), Triton-X (≥95.9%), ciprofloxacin HCl (99.7%) and 2,2-diphenyl-1-picrylhydrazyl (DPPH ≥ 99%) were directly purchased from Sigma-Aldrich (St Louis, MO, USA). Meanwhile solvents including dichloromethane (≥99.5%), 2-propanol (≥99.8%), petroleum ether (≥99%), and methanol (≥99.9%) were procured from Merck (Darmstadt, Germany). Ultra-pure water (diagnostic lab, Lahore, Pakistan) was used for extraction/dilution purposes.

### 3.2. Plant Material

The *C. sativa* leaves (CSL) were collected from Kharian, located in the Gujrat district of Punjab, Pakistan. For the botanical classification of the plant, the CSL was submitted to Dr. Tehmeena Iftikhar, Professor at Department of Botany, Government College University, Lahore, and a voucher specimen was deposited under reference number 1252. The CSL were washed with distilled water, dried under shade at room temperature (20–25 °C), grinded into coarse particles (80–100 Mesh), and stored in airtight black-colored zipper bags to protect them from light and moisture.

### 3.3. Preparation and Characterization of HDES

To synthesize HDES, menthol and thymol were used as HBA and HBD, respectively. Both components were dried separately under vacuum (0.1 bar) at low temperature (5–10 °C). These components were mixed in (1:1) molar ratios and continuously agitated until the formation of a homogenous and transparent solution. The solution was further subjected to rotary evaporation at 40 °C under reduced pressure to remove any residual free menthol, thymol, or water [22]. The resultant HDES was characterized for key intermolecular interaction by Fourier-transform Infra-red (FTIR) Spectrophotometer (IRTracer-100, Shimadzu, Kyoto, Japan).

### 3.4. Extraction Procedure

For each experimental run, exactly 2 g of powder of shade-dried *C. sativa* leaves (CSL) was taken in a conical flask (100 mL) and mixed with different ratios of HDES and ethanol, as shown in Supplementary File Table S1. Moreover, a mixture of ethanol and water was employed as a control to compare the activity with the HDES system. The extraction of bioactives was achieved through mechanical shaking briefly described as follows: a magnetic stirrer was added to the conical flask and heated with continuous stirring on the temperature-controlled hotplate, according to a varying range of temperature and time presented in Table S1. After completion of the extraction procedure, the raffinate and extracts were separated out by filtration through a filter (pore sized 1–2 microns), and the extract was evaporated in a water bath (XMTD 204). The extract was weighed to determine the bioactive yield at particular ratios of DES, ethanol, different temperatures and stirring ranges.

### 3.5. Experimental Layout and Statistical Evaluation

The factors affecting the extraction of CSL bioactives during the HDES-based extraction system were selected through initial trials and the available literature. The shaking speed (A) varied between 150 and 300 rpm, the DES-to-solid ratio (B) ranged from 1 to 10, the temperature (C) was set between 25 and 75 °C, and the extraction time (D) was tested from 70 to 170.5 min. Overall, five, eight, and eight runs were carried out at the center, factorial and axial points of a central composite design (Table S1) using response surface methodology (RSM). The amount of bioactive (g/100 g of CSL powder) was subjected to analysis of variance (ANOVA) using a statistical workstation Design Expert version 11 provided by Stat-Ease Inc., Minneapolis, MN, USA. The extraction yield was mathematically modulated following second order regression Equation (1).

(1) $$y=\beta_{o}+\in +\sum_{i=1}^{k}\beta_{i}X_{i}+\sum_{i=1}^{k}\beta_{ii}X_{i}^{2}+\Sigma_{i=1}^{k}{\sum_{j=i+1}^{k-1}\beta_{ij}X}_{i}X_{j}$$

Here y, $X_{i-j}$, $\beta_{i-j}$, and ∈ represent the extract yield (g/2 g), factors tested, regression coefficients and pure error, respectively [23].

### 3.6. Artificial Neural Network Modeling (ANN)

**Figure 7:**
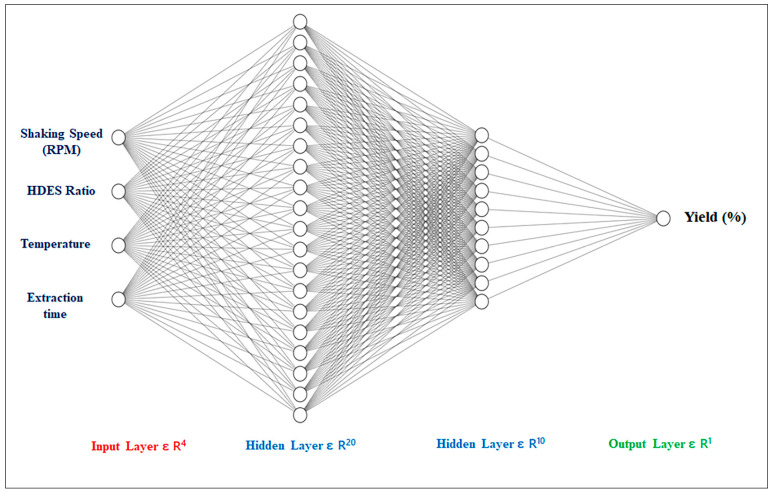
Artificial neural networking representation of four input, two hidden, and one output layers to predict CSL extract yield (g/g of CSL powder).

The experimental data obtained through Response Surface Methodology (RSM) were further used to develop predictive model using Artificial Neural Networks (ANN). The network used for ANN training was feed forward via back propagation [24]. The mean square error was used as the marker for the performance of the model. In ANN modeling, the relationship between input variables and response learnt, tested and validated during training, testing and validation, respectively, were studied, and the results are presented in Figure 7. The present model comprises 70% training and 15% each of testing and validation. It should also be mentioned here that the training session works on the basis of the Levenberg–Marquardt (LM) algorithm. The nn tool under gradient descent function mode was followed to understand the weight and bias [25]. After testing several configurations, three layers including two hidden layers (R^20^ logsig and R^10^ tansig) and one output layer (purlin) were used. All ANN modeling and simulations were performed using MATLAB R2019a with the Neural Network Toolbox [26].

### 3.7. Phenolic Content Determination of HDES Extracts

The HDES extracts of CSL obtained under optimum conditions were tested for their total phenolic content (TPC) following the method described by Waterhouse (2002) [27]. In brief, 1.0 mL of CSL extract (1.0 mg/mL) was mixed with 9.0 mL of absolute ethanol, 4.5 mL of water and 0.3 mL of 10 M Folin reagent. The whole mixture was placed in the dark for eight minutes at 25 °C. After inoculation, 900 µL of a 7.5% Na_2_CO_3_ solution was added, and the mixture was again placed in the dark at 40 °C for thirty minutes. A solution containing only the DES was used as a blank control. The UV-spectrometer at 765 nm was used for finding the absorbance. A gallic acid calibration curve was used as the standard for the quantification of TPC as milligrams of Gallic Acid Equivalents (mg GAE)/g of powdered CSL [27].

### 3.8. Flavonoids Content Determination Assay

The colorimetric method was used to determine the total flavonoids content present in CSL extracts prepared in HDES. In this method, 300 μL of each prepared extract was mixed with 3400 µL of 30% hydro-methanol in test tubes. To the resulting mixture, we added 200 μL each of 0.5 M sodium nitrite (NaNO_2_) and 0.3 M aluminum chloride (AlCl_3_), and the solution was placed in the dark for 5 min. After incubation, 150 μL of 1 Molar sodium hydroxide was added to the reaction mixture and placed in the dark for 20 min. A UV-spectrophotometer was used to measure absorbance, while rutin was used as a positive control (standard). The negative control (blank) was prepared using the HDES solution in place of the extract [28].

### 3.9. Radical Scavenging Capacity of CSL Extracts

The free radical inhibition potential of the CSL extracts obtained via HDES-based extraction was determined by following a previously reported assay [29]. To perform this, a stock solution of 2,2-diphenyl-1-picryldyrazyl (DPPH) was prepared in methanol and diluted with the same diluent until it reads 1.0 absorption at 517 nm (λ_max_). Then, 2.0 mL of CSL extract was added to an equal volume of the above DPPH solution and incubated in the dark at 37 °C for 5 min. The negative control (blank) was prepared using the HDES solution in place of the extract by following the same procedure. The absorbance of DPPH (A_c_) and sample (A_s_) was measured at 517 nm to determine the free radical scavenging activity (RSC) of CSL.

$$\%\;DPPH=100\;(\frac{A_{c}-A_{s}}{A_{c}})$$

### 3.10. Inhibition of Alpha-Amylase

The ability of CSL extracts to inhibit α-amylase was evaluated following the method described by Sharif et al. [30]. In this assay, 2 mL of α-amylase (300 units/mL) was incubated with an equal volume of CSL, as well as HDES solution in place of the extract (as blank), individually for 30 min in an incubator set at 37 °C. Following the incubation, 1.0 mL of freshly prepared starch was added to above mixture and incubated for another 5 min. Then, 1.0 mL of DNS was added to the above solution, the mixture was heated at 85 °C for 30 min, and the absorbance at 540 nm was determined to calculate the percentage inhibition of α-amylase

$$\%\;\alpha -amylase\;inhibition=100\;(\frac{A_{540\;nm\;contro}-A_{540\;nm\;sample}}{A_{540\;nm\;control}})$$

### 3.11. Inhibition of Lipase

In this assay, 100 mg of lipase (1500 units) was mixed with 50 mL of pH 8 tris-buffer and centrifuged for 10 min. In a separate 100 mL flask, 113.2 mg of para-nitrophenyl phosphate (*p*-PNP) was dissolved in 2-propanol. The stock solution of the CSL extract obtained under optimum was prepared by adding 1 g in 10 mL of DMSO, followed by serial dilutions ranging from 25 to 405 μg/mL. In the final reaction, 1.0 mL of CSL extract was incubated with same volume of enzyme solution at 37 °C for 30 min followed by the addition of 1.0 mL of the substrate; orlistat was used as the positive control, and denatured enzyme was used as the blank. After an incubation of 120 min at 37 °C, the solution was monitored at 410 to calculate the percentage inhibition of pancreatic lipase by CSL extract [31].

$$\%\;Lipase\;inhibitory\;activity=100\;(\frac{A_{c}-A_{s}}{A_{c}})$$

### 3.12. Homolytic Assay

Determination of the cytotoxicity of CSL extracts obtained while applying HDES as extraction solvent was conducted using an in vitro spectroscopic assay. Chicken erythrocytes were separated from fresh blood obtained from a licensed slaughterhouse (animals slaughtered for food purposes), and no animals were slaughtered for this study. The blood samples were collected and handled following the standard procedure under the supervision of a medical officer. The separated erythrocytes were subsequently mixed with 100 mM phosphate buffer of pH 7.4. In all the samples, 0.5 mL of HDES-based CSL extract (at different concentrations ranging from 120 to 500 µg/mL) was mixed with an equal volume of erythrocyte suspension. The mixture was held in an incubator set at 37 °C for 1.0 h and then centrifuged at 2500 rpm for 7 min. Triton X-100 was treated as the positive control, and phosphate buffer was used as the blank. The absorbance of the supernatant was measured at 541 nm using a UV–Visible spectrophotometer to determine the extent of hemolysis [31].

$$\%\;\mathrm{Hemolysis}=\frac{As-Ao}{Ac}\;\times \;100$$

### 3.13. Biofilm Inhibition Activity

The biofilm inhibition assay of the CSL extract prepared in HDES was conducted using a modified version of the method described by Shahid et al. [32]. In short, 100 µL of sterilized broth, 10 µL of bacterial strain, and 100 µL of CSL extract produced by HDES under optimum conditions were added to sterilized microwells of a microtiter plate. The standard antibiotic Ciprofloxacin was used as the positive control, whereas sterile nutrient broth was treated as the blank. The plates were incubated 35 ± 5 °C for 18 h, and then, the contents of each well were washed with phosphate buffer. Finally, 99.9% methanol was added to each well followed by 15 min of incubation to fix the biofilm. The wells were kept under vacuum to remove methanol and then stained with 7% crystal violet and incubated for an additional 10 min. Excess stain was rinsed off using 200 µL of distilled water, followed by the addition of 200 µL of 33% glacial acetic acid to solubilize the bound stain. The absorbance was recorded at 620 nm using a microplate reader [33]. The percentage of biofilm inhibition was calculated using the formula given in Section 3.11.

### 3.14. GC-MS Analysis

GC-MS analysis of the CSL extract was conducted by following the reported procedures with some modifications [34]. Briefly, the HDES extracts of CSL produced under optimum conditions were mixed with an equal volume of dimethyl ether, and 0.5 µL of the upper layer was promptly injected into a gas chromatograph Agilent 8890 GC system (Agilent technologies, Santa Clara, CA, USA) equipped with an Agilent column DB-5 ms (5% phenyl-methylpolysiloxane) of dimensions 30 m × 0.32 mm × 0.25 µm, ramped from 60 to 325 °C at the rate of 10 °C/min, with the total gas flow maintained at 1.5 mL/min and the pressure at 1.995 psi. The column oven temperature was set to 50 °C for the initial 5 min, followed by a temperature ramp rate of 10 °C per minute and a final hold up time of 2 min. The injector temperature was maintained at 210.0 °C, and the injection was performed in split mode with a split ratio of 70:30. The flow control was pressure-based, set at 1.995 psi, with a column flow rate of 1.5 mL/min, a linear velocity of 44.65 cm/sec, and a purge flow rate of 3.0 mL/min. Compound detection and quantification were conducted using a single quadrupole mass detector (Agilent 5799 MSD) equipped with an electron impact (EI) ion source operating in total ion current (TIC) mode. The cut-off of 2 min and temperature of 210 °C with scan acquisition mode of *m*/*z* 50.0 to 550 amu and a scan speed 2.9 scans/sec are the specifications of GC-MS.

## 4. Conclusions

The present study has been undertaken to evaluate the effectiveness of a hydrophobic deep eutectic solvent (HDES) to extract bioactive compounds present in *Cannabis Sativa* L. leaves (CSL) without compromising their antioxidant potential. The observed trend of extraction yield, total phenolic content (TPC), total flavonoid content (TFC), free radical scavenging potential, antidiabetic activity, and hemolytic safety indicates the HDES in combination with ethanol can work as an efficient sustainable extraction solvent. The observed data further indicates the HDES–ethanol system significantly improved the recovery of TPC (133.75 ± 1.156 mg GAE/g) and TFC (108.05 ± 8.89 mg QE/g), far exceeding conventional extraction mediums. Bioactivity assays confirmed a strong antioxidant potential (via the DPPH assay) and dose-dependent enzyme inhibition, including α-amylase (up to 68.8%) and lipase activity (23.6%), indicating potential antidiabetic and anti-obesity effects. The extracts demonstrated mild hemolytic activity, confirming low toxicity and moderate biofilm inhibition against both *S. aureus* and *S. epidermidis*. GC-MS analysis identified key cannabinoids (cannabidiol, cannabinol, cannabidivarol and tetrahydrocannabivarin), terpenoids (dl-menthol, levomenthol), and the dominant antioxidant compound 4-hydroxy-3-methylacetophenone, which could be responsible for key therapeutic values. In conclusion, HDES–ethanol extraction offers a green, efficient, and biocompatible approach for isolating bioactive compounds from *C. sativa*, with promising applications in pharmaceuticals targeting oxidative stress, metabolic disorders, and microbial infections.

## Figures and Tables

**Table 1:** The analysis of variance (ANOVA) data regarding HDES-based extraction of CSL bioactives.

	Source	Sum of Squares	df	Mean Square	F-Value	* *p*-Value
	Model	3.49	14	0.2493	59.89	<0.0001
Linear Effects	A	0.1250	1	0.1250	30.03	0.0015
	B	0.2178	1	0.2178	52.32	0.0004
	C	0.0818	1	0.0818	19.64	0.0044
	D	0.2048	1	0.2048	49.20	0.0004
Interaction	AB	0.1317	1	0.1317	31.64	0.0014
	AC	0.1636	1	0.1636	39.30	0.0008
	AD	0.1618	1	0.1618	38.86	0.0008
	BC	0.0082	1	0.0082	1.97	0.2102
	BD	0.1519	1	0.1519	36.49	0.0009
	CD	0.0283	1	0.0283	6.80	0.0402
Quadratic Effects	A^2^	0.3626	1	0.3626	87.10	<0.0001
	B^2^	0.3285	1	0.3285	78.93	0.0001
	C^2^	0.0764	1	0.0764	18.36	0.0052
	D^2^	0.0725	1	0.0725	17.41	0.0059
**Residuals**		0.0250	6	0.0042		
Lack of Fit		0.0173	2	0.0086	4.50	0.0946
Pure Error		0.0077	4	0.0019		
**Corrected Total**		3.52	20			

* Probability (*p)* ≤ 0.05 indicates significant differences. A, B, C, and D are the labels used shaking speed, DES ratio, temperature, and extraction time, respectively.

**Table 2:** Yield comparison of actual and predicted values generated by experimental analysis along with RSM and ANN predictions.

Std	Run	A: Shaking Speed	B: DES	C: Temperature	D: time	Yield * Experimental	Yield * RSM Predicted	Yield * ANN Predicted
		Rpm	Ratio	°C	Min			
20	1	225	5.5	55	107.5	1.30	1.20	1.23
6	2	175	2.5	75	82.5	0.05	0.03	0.04
5	3	275	2.5	35	132.5	0.02	0.00	0.02
15	4	225	5.5	55	70	1.30	1.34	1.30
19	5	225	5.5	55	107.5	1.20	1.20	1.23
9	6	150	5.5	55	107.5	0.50	0.54	0.50
2	7	275	8.5	35	82.5	1.12	1.10	1.12
1	8	275	8.5	75	82.5	1.06	1.04	1.06
7	9	175	8.5	75	132.5	1.02	1.00	1.02
4	10	175	8.5	35	132.5	1.41	1.39	1.41
21	11	225	5.5	55	107.5	1.20	1.20	1.23
8	12	175	2.5	35	82.5	0.55	0.53	0.55
3	13	275	2.5	75	132.5	0.33	0.31	0.37
18	14	225	5.5	55	107.5	1.20	1.20	1.23
10	15	300	5.5	55	107.5	1.00	1.04	1.00
12	16	225	10	55	107.5	1.10	1.14	1.08
17	17	225	5.5	55	107.5	1.24	1.20	1.23
14	18	225	5.5	85	107.5	0.85	0.89	0.85
16	19	225	5.5	55	145	0.66	0.70	0.66
11	20	225	1	55	107.5	0.44	0.48	0.44
13	21	225	5.5	25	107.5	1.10	1.13	1.10

* g/2g of shade dried CSL powder.

**Table 3:** Comparison of the statistical performance metrics including coefficient of determination (R^2^), absolute average deviation (AAD), percentage prediction error (PPE), and root mean square error (RMSE) obtained from the RSM and ANN models.

Parameters	Yield	
	RSM	ANN
R^2^	0.9925	0.9952
AAD (%)	9.85	2.30
PPE (%)	8.50	4.99
RMSE	0.035	0.029

**Table 4:** Relationship between experimental value of yield and RSM and ANN predicted values.

Responses	Experimental	Predicted	
		RSM	ANN
Yield (g/2 g of CSL)	0.871 ± 0.19	0.945	0.828

**Table 5:** Total phenolic and total flavonoid content of CSL extracts produced via HDES.

Solvent	Conditions	TPC (mg GAE/g)	TFC (mg/QE/g)	Reference
5.5 mL DES + 4.5 ethanol (Solution 1)	Shaking speed 225 rpm, 55 °C, shaking time 107.5 min	133.07 ± 1.87	108.05 ± 8.89	Present study
8.5 mL DES + 1.5 ethanol (Solution 2)	Shaking speed 175 rpm, 35 °C, shaking time 132.5 min	108.20 ± 4.23	55.05 ± 2.05	
Ethanol	Shaking speed 225 rpm, 55 °C, shaking time 10.75 min	9.52 ± 0.23	4.12 ± 0.17	
Water	Blend in water made at shaking speed 225 rpm, 55 °C and for 10.75 min	3.26 ± 1.02	3.04 ± 1.01	
Ethanol/water 50/50 (*v*/*v*)	Shaking at 25 °C for 24 h	9.25	5.21	[18]
Ethanol/water 90/10 (*v*/*v*)		5.85	3.18	
Pure water		6.21	1.83	
Methanol	Soaking for 72 h at 25 °C	36.42	59	[19]
Ethanol		2.70	56	
Ethyl acetate		12.16	ND	
Chloroform		ND	ND	
Water		29.98	ND	

**Table 6:** Bioactive compounds identified in hydrophobic deep eutectic solvent (HDES)-based extracts of *Cannabis sativa* leaf (CSL) during gas chromatography–mass spectrometry (GC–MS) analysis.

Rt min	M.W. (g mol^−1^)	Area (%)	CAS #	Library Score	Compound Name
4.174	204.35	0.01	87-44-5	97	β-Caryophyllene
4.932	136.24	0.06	5989-27-5	99	Limonene
6.184	154.25	0.01	106-24-1	64	Geraniol
7.252	154.25	0.15	14073-97-3	93	L-Menthone
7.473	152.23	0.05	76-22-2	93	Camphor
8.362	156.27	19.93	89-78-1	91	dl-Menthol
8.503	156.27	9.67	2216-51-5	91	Levomenthol
8.717	156.27	4.87	2216-51-5	91	Menthol
10.339	164.204	0.01	6138-88-1	83	Dehydroelsholtzia ketone
12.468	302.27	46.48	520-26-3	70	Hesperidin
12.62	150.22	0.19	4167-74-2	90	p-Isobutylphenol
12.852	150.22	0.02	89-83-8	90	Thymol
13.018	196.29	0.04	105-87-3	93	Geranyl Acetate
13.407	150.22	0.03	3228-02-02	60	p-Cymen-5-ol
13.698	164.2	0.04	97-53-0	74	Eugenol
13.916	314.5	0.02	21366-63-2	91	Cannabicyclol
14.519	204.36	0.04	87-44-5	99	Caryophyllene
14.638	150.22	0.02	89-83-8	91	Thymol
14.762	204.35	0.01	28973-99-1	70	(Z, Z)-α-Farnesene
15.139	204.35	0.02	495-61-4	98	β-Bisabolene
15.454	204.35	0.01	17066-67-0	80	β-Selinene
15.681	204.35	0.01	6753-98-6	99	Alpha-Humulene
15.791	220.35	0.01	1139-30-6	95	Caryophyllene oxide
16.586	166.217	0.05	2217-60-9	95	p-Cymene diol
17.043	220.35	0.01	1139-30-6	76	Caryophyllene Oxide
17.986	204.35	0.007	29873-99-2	64	Gamma-Elemene
18.355	222.37	0.008	23089-26-1	68	Levomenol
20.064	278.52	0.01	504-96-1	99	Neophytadiene
24.291	296.5	0.02	7541-49-3	86	Phytol
26.445	286.4	0.05	24274-48-4	99	Cannabidivarin
27.16	220.33	0.01	1139-30-6	55	α-Caryophyllene oxide
27.572	314.46	0.01	13956-29-1	87	Cannabidiol
27.572	314.5	0.01	20675-51-8	87	Cannabichromene
28.061	286.46	0.34	31262-37-0	99	Tetrahydrocannabivarin
28.644	246.302	0.02	61262-81-5	95	Cannabispiran
28.844	136.22	0.09	13466-78-9	94	Delta-3-Carene
29.926	222.37	0.02	1139-17-9	64	Isolongifolol
30.171	314.5	0.01	5957-75-5	64	Δ8-THC
30.574	314.47	0.63	1972-08-03	99	Dronabinol
31.192	310.4	0.17	521-35-7	99	Cannabinol
32.337	282.56	0.01	112-95-8	97	Eicosane
33.924	254.48	0.03	593-45-3	97	Octadecane
37.047	414.72	0.01	83-46-5	91	Beta-Sitosterol
37.944	220.34	0.01	128-37-0	56	Butylated Hydroxytoluene

## Data Availability

The original contributions presented in this study are included in the article/Supplementary Material. Further inquiries can be directed at the corresponding authors.

## Supplementary Materials

The following supporting information can be downloaded at https://www.mdpi.com/article/10.3390/ijms27072933/s1.
