# Supplementary material for: Natural Hydrophobic Deep Eutectic Solvent-Based Enhanced Extraction of Bioactive Compounds from Cannabis sativa L. Leaf for Pharmaceutical Applications
Source: Int J Mol Sci. 2026 Mar 24;27(7):2933. doi: 10.3390/ijms27072933 (PMC13073703; doi:10.3390/ijms27072933)
Supplement: Supplementary file 1 [file ijms-27-02933-s001.zip › ijms-4121792-supplementary.pdf]

Natural Hydrophobic Deep Eutectic Solvent-Based Enhanced Extraction of Bioactive Compounds from *Cannabis sativa* L. Leaf for Pharmaceutical Applications

Serwat Naz [1], Sumia Akram [2], Rabia Naeem [1], Haroon Iftikhar [1], Rizwan Ashraf [3], Noor Ul Ain Khalid [1], Muhammad Shahid [4], Imad A. Abu-Yousef [5]*, Amin F. Majdalawieh [5, 6, 7] and Muhammad Mushtaq [1]*

[1] Department of Chemistry, Government College University Lahore, Lahore 54000, Pakistan; serwat432@gmail.com (S.N.); rabianaeem@gcu.edu.pk (R.N.); chemistharoon@yahoo.com (H.I.); chemistnoor@yahoo.com (N.-U.-A.K.)

[2] Division of Science and Technology, University of Education Lahore, Lahore 54770, Pakistan; sumia.akram@ue.edu.pk

[3] Department of Chemistry, University of Agriculture, Faisalabad 38000, Pakistan; rizi_chem82@hotmail.com

[4] Department of Biochemistry, University of Agriculture, Faisalabad 38000, Pakistan; mshahiduaf@yahoo.com

[5] Department of Biology, Chemistry and Environmental Sciences, College of Arts and Sciences, American University of Sharjah, Sharjah P.O. Box 26666, United Arab Emirates; amajdalawieh@aus.edu

[6] Advanced Biosciences and Bioengineering Research Center, American University of Sharjah, Sharjah, P.O. Box 26666, United Arab Emirates

[7] Bioinformatics and Computational Biology Research Group, American University of Sharjah, Sharjah, P.O. Box 26666, United Arab Emirates

* Correspondence: iabuyousef@aus.edu (I.A.A.-Y.); muhammad.mushtaq@gcu.edu.pk (M.M.)

Table S1. % Yield comparison of actual, and predicted values generated by experimental analysis along with RSM and ANN predictions

Std	Run	A: shaking speed	B: Ratio	C: Temperature	D: time	% Yield Experimental	% Yield RSM predicted	% Yield ANN predicted
		rpm	DES/Ethanol	°C	min			
20	1	225	5.5	55	107.5	1.30	1.20	1.23

6	2	175	2.5	75	82.5	0.05	0.03	0.04
5	3	275	2.5	35	132.5	0.02	0.00	0.02
15	4	225	5.5	55	70	1.30	1.34	1.30
19	5	225	5.5	55	107.5	1.20	1.20	1.23
9	6	150	5.5	55	107.5	0.50	0.54	0.50
2	7	275	8.5	35	82.5	1.12	1.10	1.12
1	8	275	8.5	75	82.5	1.06	1.04	1.06
7	9	175	8.5	75	132.5	1.02	1.00	1.02
4	10	175	8.5	35	132.5	1.41	1.39	1.41
21	11	225	5.5	55	107.5	1.20	1.20	1.23
8	12	175	2.5	35	82.5	0.55	0.53	0.55
3	13	275	2.5	75	132.5	0.33	0.31	0.37
18	14	225	5.5	55	107.5	1.20	1.20	1.23
10	15	300	5.5	55	107.5	1.00	1.04	1.00
12	16	225	10	55	107.5	1.10	1.14	1.08
17	17	225	5.5	55	107.5	1.24	1.20	1.23
14	18	225	5.5	85	107.5	0.85	0.89	0.85
16	19	225	5.5	55	145	0.66	0.70	0.66
11	20	225	1	55	107.5	0.44	0.48	0.44
13	21	225	5.5	25	107.5	1.10	1.13	1.10

The design matrix was generated following a partially rotatable central composite design (α=1.5), std indicates standard randomization order.

Table S2 : GC-MS library match and predicted chemical identity of components

Library Search Report

Data Path : C:\GCMS Data\05-06-2024\
Data File : S678.D
Acq On : 06 Jun 2024 19:14
Operator :
Sample : S-6, 7, 8
Misc :
ALS Vial : 7 Sample Multiplier: 1

Search Libraries: C:\MassHunter\LIBRARY\NIST20.L Minimum Quality: 0

Unknown Spectrum: Apex
Integration Events: ChemStation Integrator - autoint1.e

Pk#	RT	Area%	Library/ID	Ref#	CAS#	Qual
1	4.174	0.01	C:\MassHunter\LIBRARY\NIST20.L			
			Bicyclo[3.1.0]hexane, 4-methylene-1-(1-methylethyl)-	18240	003387-41-5	97
			Cyclohexene, 4-methylene-1-(1-methylethyl)-	18182	000099-84-3	91
			Bicyclo[3.1.0]hexane, 4-methylene-1-(1-methylethyl)-	18245	003387-41-5	91
2	4.360	0.00	C:\MassHunter\LIBRARY\NIST20.L			
			.beta.-Myrcene	18031	000123-35-3	93
			.beta.-Pinene	18023	000127-91-3	87
			Bicyclo[3.1.0]hexane, 4-methylene-1-(1-methylethyl)-	18243	003387-41-5	86
3	4.458	0.00	C:\MassHunter\LIBRARY\NIST20.L			
			3-Octanol	15662	000589-98-0	72
			3-Octanol	15663	000589-98-0	72
			3-Octanol	15660	000589-98-0	72
4	4.932	0.06	C:\MassHunter\LIBRARY\NIST20.L			
			D-Limonene	18009	005989-27-5	99
			D-Limonene	18005	005989-27-5	94
			Limonene	17995	000138-86-3	91
5	5.144	0.00	C:\MassHunter\LIBRARY\NIST20.L			
			.beta.-Ocimene	18026	013877-91-3	97
			1,3,6-Octatriene, 3,7-dimethyl-, (Z)-	18141	003338-55-4	97
			1,3,6-Octatriene, 3,7-dimethyl-, (Z)-	18140	003338-55-4	90
6	5.923	0.04	C:\MassHunter\LIBRARY\NIST20.L			
			Propane, 2-fluoro-2-methyl-	1114	000353-61-7	9
			Glycerin	2719	000056-81-5	9
			Methanamine, N-methoxy-	360	001117-97-1	5
7	6.184	0.01	C:\MassHunter\LIBRARY\NIST20.L			
			Pyrimidine, 2-ethoxy-4,6-dimethyl-	29890	007781-21-7	64
			Acetaldehyde, (3,3-dimethylcyclohexylidene)-, (E)-	29363	026532-25-2	55
			(3E,5E)-2,6-Dimethylocta-3,5,7-trien-2-ol	29282	206115-88-0	46
8	7.070	0.13	C:\MassHunter\LIBRARY\NIST20.L			
			Bicyclo[2.2.1]heptan-2-one, 1,7,7-trimethyl-, (1S)-	29380	000464-48-2	98
			(+)-2-Bornanone	29115	000464-49-3	97
			(+)-2-Bornanone	29114	000464-49-3	96
9	7.252	0.15	C:\MassHunter\LIBRARY\NIST20.L			
			Bicyclo[2.2.1]heptan-2-one, 1,7,7-trimethyl-, (1S)-	29381	000464-48-2	93
			L-Menthone	30920	014073-97-3	83
			Camphor	29049	000076-22-2	83

Library Search Report

Data Path : C:\GCMS Data\05-06-2024\
Data File : S678.D
Acq On : 06 Jun 2024 19:14
Operator :
Sample : S-6, 7, 8
Misc :
ALS Vial : 7 Sample Multiplier: 1

Search Libraries: C:\MassHunter\LIBRARY\NIST20.L Minimum Quality: 0

Unknown Spectrum: Apex
Integration Events: ChemStation Integrator - autoint1.e

Pk#	RT	Area%	Library/ID	Ref#	CAS#	Qual
10	7.473	0.05	C:\MassHunter\LIBRARY\NIST20.L			
			Bicyclo[2.2.1]heptan-2-one, 1,7,7-trimethyl-, (1S)-	29373	000464-48-2	93
			Bicyclo[2.2.1]heptan-2-one, 1,7,7-trimethyl-, (1S)-	29381	000464-48-2	93
			Camphor	29042	000076-22-2	93
11	7.610	0.09	C:\MassHunter\LIBRARY\NIST20.L			
			Cyclohexane, 1-methyl-4-(1-methylethylidene)-	19538	001124-27-2	93
			Bicyclo[4.1.0]heptane, 3,7,7-trimethyl-, (1.alpha.,3.alpha.,6.alpha.)-	19560	018968-23-5	92
			Bicyclo[4.1.0]heptane, 3,7,7-trimethyl-, [1S-(1.alpha.,3.beta.,6.alpha.)]-	19563	002778-68-9	70
12	8.362	19.93	C:\MassHunter\LIBRARY\NIST20.L			
			dl-Menthol	32850	000089-78-1	91
			Levomenthol	32859	002216-51-5	91
			dl-Menthol	32851	000089-78-1	91
13	8.503	9.67	C:\MassHunter\LIBRARY\NIST20.L			
			Levomenthol	32859	002216-51-5	91
			dl-Menthol	32851	000089-78-1	91
			dl-Menthol	32850	000089-78-1	91
14	8.717	4.87	C:\MassHunter\LIBRARY\NIST20.L			
			dl-Menthol	32850	000089-78-1	91
			Cyclohexanol, 5-methyl-2-(1-methylethyl)-	32983	001490-04-6	91
			dl-Menthol	32851	000089-78-1	91
15	8.817	15.80	C:\MassHunter\LIBRARY\NIST20.L			
			dl-Menthol	32851	000089-78-1	91
			Levomenthol	32859	002216-51-5	91
			dl-Menthol	32850	000089-78-1	91
16	10.339	0.01	C:\MassHunter\LIBRARY\NIST20.L			
			Dehydroelsholtzia ketone	39249	006138-88-1	83
			2,3-Dimethyltricyclo[2.2.1.02,6]heptane-3-carboxylic acid	41300	000562-66-3	44
			2-(1-Hydroxybut-2-enylidene)cyclohexanone	41224	1000186-18-9	42
17	12.468	46.48	C:\MassHunter\LIBRARY\NIST20.L			
			4-Hydroxy-3-methylacetophenone	28353	000876-02-8	70
			4-Hydroxy-3-methylacetophenone	28357	000876-02-8	70
			2,5-Diethylphenol	27461	000876-20-0	68
18	12.620	0.19	C:\MassHunter\LIBRARY\NIST20.L			
			2-Methyl-6-propylphenol	27487	003520-52-3	90
			Phenol, 4-(1-methylpropyl)-	27512	000099-71-8	86

Library Search Report

```
Data Path : C:\GCMS Data\05-06-2024\
Data File : S678.D
Acq On    : 06 Jun 2024  19:14
Operator  :
Sample    : S-6, 7, 8
Misc      :
ALS Vial  : 7   Sample Multiplier: 1

Search Libraries:   C:\MassHunter\LIBRARY\NIST20.L    Minimum Quality:   0

Unknown Spectrum:   Apex
Integration Events: ChemStation Integrator - autoint1.e

Pk#     RT  Area%          Library/ID                 Ref#     CAS#    Qual
_____________________________________________________________________________
               Phenol, 2-(1-methylpropyl)-         27504 000089-72-5 80

 19  12.852  0.02 C:\MassHunter\LIBRARY\NIST20.L
               Phenol, 2-methyl-5-(1-methylethyl)  27579 000499-75-2 90
               3-Methyl-4-isopropylphenol          27496 003228-02-2 87
               Phenol, 2-methyl-5-(1-methylethyl)  27581 000499-75-2 87

 20  13.018  0.04 C:\MassHunter\LIBRARY\NIST20.L
               Phenol, 5-methyl-2-(1-methylethyl)  66733 000528-79-0 93
               -, acetate
               Phenol, 2-ethyl-4,5-dimethyl-       27532 002219-78-5 76
               Phenol, 5-methyl-2-(1-methylethyl)  66732 000528-79-0 76
               -, acetate

 21  13.407  0.03 C:\MassHunter\LIBRARY\NIST20.L
               3-tert-Butyl-2-hydroxybenzaldehyde  52595 024623-65-2 60
               6-tert-Butyl-2,4-dimethylphenol     52844 001879-09-0 58
               6-tert-Butyl-2,4-dimethylphenol     52840 001879-09-0 49

 22  13.698  0.04 C:\MassHunter\LIBRARY\NIST20.L
               2-Methoxybenzoic acid, allyl ester  66356 031994-79-3 74
               1-(2-Methyl-4-propoxy-phenyl)-etha  66674 1000187-58-2 70
               none
               3-(2-Methoxy-5-methylphenyl)propio  68801 061371-44-6 70
               nic acid

 23  13.916  0.02 C:\MassHunter\LIBRARY\NIST20.L
               Benzene, 1,2,4,5-tetraethyl-        64876 000635-81-4 91
               2-Acetyl-3-methylbenzo[b]thiophene  64489 018781-31-2 78
               2-Acetyl-4-methylbenzo(b)thiophene  64490 001467-88-5 60

 24  14.095  0.02 C:\MassHunter\LIBRARY\NIST20.L
               8,9-Dehydrothymol                   26187 018612-99-2 96
               Benzene, 1-ethyl-3-(1-methylethyl)  26395 004920-99-4 76
               Benzene, 1-methoxy-2-(1-methylethe  26313 010278-02-1 74
               nyl)-

 25  14.519  0.04 C:\MassHunter\LIBRARY\NIST20.L
               Caryophyllene                       80121 000087-44-5 99
               Bicyclo[5.2.0]nonane, 2-methylene-  80294 242794-76-9 98
               4,8,8-trimethyl-4-vinyl-
               Caryophyllene                       80124 000087-44-5 96

 26  14.638  0.02 C:\MassHunter\LIBRARY\NIST20.L
               5-Methyl-2,4-diisopropylphenol      66876 040625-96-5 91
               5-Methyl-2,4-diisopropylphenol      66875 040625-96-5 87
               1,3-Benzodioxol-2-one, 5-(1,1-dime  66411 054815-21-3 76
               thylethyl)-

 27  14.762  0.01 C:\MassHunter\LIBRARY\NIST20.L
               (Z,Z)-.alpha.-Farnesene             80225 1000293-03-1 70
               Bicyclo[3.1.1]hept-2-ene, 2,6-dime  80319 017699-05-7 70
               thyl-6-(4-methyl-3-pentenyl)-
               1,3,6,10-Dodecatetraene, 3,7,11-tr  80268 026560-14-5 70
```

Library Search Report

```
Data Path : C:\GCMS Data\05-06-2024\
Data File : S678.D
Acq On    : 06 Jun 2024  19:14
Operator  :
Sample    : S-6, 7, 8
Misc      :
ALS Vial  : 7   Sample Multiplier: 1

Search Libraries:   C:\MassHunter\LIBRARY\NIST20.L    Minimum Quality:   0

Unknown Spectrum:   Apex
Integration Events: ChemStation Integrator - autoint1.e

Pk#     RT  Area%          Library/ID                 Ref#     CAS#   Qual
_____________________________________________________________________________
                 imethyl-, (Z,E)-

 28  14.928  0.00 C:\MassHunter\LIBRARY\NIST20.L
                 Aromandendrene                        80136 000489-39-4 55
                 Benzamide, 3-methoxy-N-methyl-N-pr    83785 1000421-51-8 53
                 opyl-
                 Benzeneacetaldehyde, 2-methoxy-       28361 033567-59-8 50

 29  15.139  0.02 C:\MassHunter\LIBRARY\NIST20.L
                 1,4,7,-Cycloundecatriene, 1,5,9,9-    80275 1000062-61-9 98
                 tetramethyl-, Z,Z,Z-
                 Humulene                              80087 006753-98-6 97
                 Humulene                              80088 006753-98-6 96

 30  15.365  0.00 C:\MassHunter\LIBRARY\NIST20.L
                 Cycloheptasiloxane, tetradecamethy   340529 000107-50-6 64
                 l-
                 Cycloheptasiloxane, tetradecamethy   340530 000107-50-6 64
                 l-
                 Benzamide, 3-methoxy-N-[4-(1-methy   174740 1000351-11-1 46
                 lcyclopropyl)phenyl]-

 31  15.454  0.01 C:\MassHunter\LIBRARY\NIST20.L
                 (S,1Z,6Z)-8-Isopropyl-1-methyl-5-m    80314 317819-80-0 80
                 ethylenecyclodeca-1,6-diene
                 Naphthalene, 1,2,4a,5,6,8a-hexahyd    80351 000483-75-0 52
                 ro-4,7-dimethyl-1-(1-methylethyl)-
                 Naphthalene, decahydro-1,6-bis(met    80462 030021-46-6 51
                 hylene)-4-(1-methylethyl)-, (4.alp
                 ha.,4a.alpha.,8a.alpha.)-

 32  15.681  0.01 C:\MassHunter\LIBRARY\NIST20.L
                 Naphthalene, decahydro-4a-methyl-1    80481 017066-67-0 99
                 -methylene-7-(1-methylethenyl)-, [
                 4aR-(4a.alpha.,7.alpha.,8a.beta.)]
                 .beta.-Humulene                       80147 000116-04-1 97
                 Naphthalene, decahydro-4a-methyl-1    80473 017066-67-0 96
                 -methylene-7-(1-methylethenyl)-, [
                 4aR-(4a.alpha.,7.alpha.,8a.beta.)]

 33  15.791  0.01 C:\MassHunter\LIBRARY\NIST20.L
                 Naphthalene, decahydro-4a-methyl-1    80431 000515-17-3 95
                 -methylene-7-(1-methylethylidene)-
                 , (4aR-trans)-
                 1H-Cycloprop[a]naphthalene, decah    80537 020071-49-2 91
                 ydro-1,1,3a-trimethyl-7-methylene-
                 , [1aS-(1a.alpha.,3a.alpha.,7a.bet
                 a.,7b.alpha.)]-
                 Spiro[5.5]undec-2-ene, 3,7,7-trime    80303 018431-82-8 90
                 thyl-11-methylene-, (-)-

 34  16.055  0.01 C:\MassHunter\LIBRARY\NIST20.L
                 Aromandendrene                        80135 000489-39-4 25
                 1H-Pyrazol-5-amine, 3-methyl-1-phe    48232 001131-18-6 15
```

Library Search Report

```
Data Path : C:\GCMS Data\05-06-2024\
Data File : S678.D
Acq On    : 06 Jun 2024  19:14
Operator  :
Sample    : S-6, 7, 8
Misc      :
ALS Vial  : 7   Sample Multiplier: 1

Search Libraries:   C:\MassHunter\LIBRARY\NIST20.L    Minimum Quality:   0

Unknown Spectrum:   Apex
Integration Events: ChemStation Integrator - autoint1.e

Pk#     RT  Area%          Library/ID                Ref#     CAS#    Qual
___________________________________________________________________________
                 nyl-
                 4-(2-Hydroxyethyl)-5-oxo-3-phenyl-  79534 010244-77-6 15
                 2-pyrazoline

 35  16.443  0.00 C:\MassHunter\LIBRARY\NIST20.L
                 Cyclohexene, 4-[(1E)-1,5-dimethyl-  80317 025532-79-0 97
                 1,4-hexadien-1-yl]-1-methyl-
                 Bicyclo[2.2.1]heptane, 7,7-dimethy  18226 000471-84-1 60
                 l-2-methylene-
                 cis-.alpha.-Bisabolene              80218 029837-07-8 58

 36  16.586  0.05 C:\MassHunter\LIBRARY\NIST20.L
                 p-Cymene-2,5-diol                   41078 002217-60-9 95
                 Ethanone, 1-(2-hydroxy-6-methoxyph  42248 000703-23-1 86
                 enyl)-
                 Ethanone, 1-(2-hydroxy-6-methoxyph  42256 000703-23-1 86
                 enyl)-

 37  17.043  0.01 C:\MassHunter\LIBRARY\NIST20.L
                 Caryophyllene oxide                 99191 001139-30-6 76
                 2-((2R,4aR,8aS)-4a-Methyl-8-methyl  99331 000515-20-8 76
                 enedecahydronaphthalen-2-yl)prop-2
                 -en-1-ol
                 1-Methyl-6-(3-methylbuta-1,3-dieny  52943 1000185-67-2 70
                 l)-7-oxabicyclo[4.1.0]heptane

 38  17.410  0.01 C:\MassHunter\LIBRARY\NIST20.L
                 Tricyclo[4.4.1.0(1,6)]undecane      27751 006571-73-9 42
                 3-Methoxybenzyl alcohol             19909 006971-51-3 35
                 1H-3a,7-Methanoazulene, 2,3,6,7,8,  80506 000560-32-7 30
                 8a-hexahydro-1,4,9,9-tetramethyl-,
                  (1.alpha.,3a.alpha.,7.alpha.,8a.b
                 eta.)-

 39  17.659  0.01 C:\MassHunter\LIBRARY\NIST20.L
                 1-Pyridin-3-yl-1,4-diazepane        51755 223796-20-1 38
                 6-Acetaminoflavanone               174630 1000423-07-5 38
                 Spiro[benzo-1,3-dioxolane-2,3'-pyr  51711 024476-94-6 18
                 rolidine]

 40  17.986  0.00 C:\MassHunter\LIBRARY\NIST20.L
                 .gamma.-Elemene                     80157 029873-99-2 64
                 .gamma.-Elemene                     80148 029873-99-2 64
                 .beta.-Humulene                     80147 000116-04-1 59

 41  18.174  0.00 C:\MassHunter\LIBRARY\NIST20.L
                 4-Hydroxy-3-methylacetophenone      28353 000876-02-8 38
                 p-Cymen-7-ol                        27440 000536-60-7 30
                 Icosa-9,11-diyne                   166403 028393-07-9 30

 42  18.355  0.00 C:\MassHunter\LIBRARY\NIST20.L
                 Levomenol                          101922 023089-26-1 68
                 .alpha.-Bisabolol                  101962 000515-69-5 64
                 (1S,2R,5R)-2-Methyl-5-((R)-6-methy 102057 058319-05-4 58
```

Library Search Report

```
Data Path : C:\GCMS Data\05-06-2024\
Data File : S678.D
Acq On    : 06 Jun 2024  19:14
Operator  :
Sample    : S-6, 7, 8
Misc      :
ALS Vial  : 7   Sample Multiplier: 1

Search Libraries:   C:\MassHunter\LIBRARY\NIST20.L    Minimum Quality:   0

Unknown Spectrum:   Apex
Integration Events: ChemStation Integrator - autoint1.e

Pk#     RT  Area%          Library/ID                Ref#     CAS#   Qual
_________________________________________________________________________
                 lhept-5-en-2-yl)bicyclo[3.1.0]hexa
                 n-2-ol

 43  19.492  0.01 C:\MassHunter\LIBRARY\NIST20.L
                 Tricyclo[3.3.1.1(3,7)]decane-1-met 147143 1000337-10-7 58
                 hanamine, N-(4-fluorophenyl)-
                 Fumaric acid, 2-methoxyphenyl dode 294103 1000405-94-1 43
                 c-2-en-1-yl ester
                 Succinic acid, dec-2-yl 3-methoxyp 273512 1000390-98-5 43
                 henyl ester

 44  20.064  0.01 C:\MassHunter\LIBRARY\NIST20.L
                 Neophytadiene                      171299 000504-96-1 99
                 Bicyclo[3.1.1]heptane, 2,6,6-trime  19511 000473-55-2 50
                 thyl-
                 3,7,11,15-Tetramethyl-2-hexadecen- 194565 102608-53-7 46
                 1-ol

 45  20.341  0.00 C:\MassHunter\LIBRARY\NIST20.L
                 10.alpha.-Eremophilane              85361 003242-05-5 38
                 Naphthalene, decahydro-1,8a-dimeth  85389 015404-63-4 38
                 yl-7-(1-methylethyl)-, [1R-(1.alph
                 a.,4a.beta.,7.beta.,8a.alpha.)]-
                 Tricyclo[4.4.1.0(1,6)]undecane      27751 006571-73-9 30

 46  20.553  0.00 C:\MassHunter\LIBRARY\NIST20.L
                 3-Chloropropionic acid, undec-2-en 148058 1000299-21-9 58
                 yl ester
                 2-Decylfuran                        85239 083469-85-6 38
                 cis-3-Methyl-endo-tricyclo[5.2.1.0  27784 1000215-29-0 30
                 (2.6)]decane

 47  21.579  0.01 C:\MassHunter\LIBRARY\NIST20.L
                 n-Hexadecanoic acid                143510 000057-10-3 99
                 n-Hexadecanoic acid                143511 000057-10-3 96
                 n-Hexadecanoic acid                143507 000057-10-3 95

 48  22.201  0.00 C:\MassHunter\LIBRARY\NIST20.L
                 1-[4-Acetyl-1-(2-amino-4,5-dimethy 197109 1000300-66-6 64
                 l-phenyl)-2,5-dimethyl-1H-pyrrol-3
                 -yl]-ethanone
                 4-Isopropylbenzenethiol, S-pentafl 195987 1000353-28-9 53
                 uoropropionyl-
                 Flavone, 5-hydroxy-7,8-dimethoxy-  196657 003570-62-5 43

 49  22.627  0.00 C:\MassHunter\LIBRARY\NIST20.L
                 p-Acetophenetide, o-amino, N,N'-bi 101255 1000511-93-8 38
                 s-methyl
                 O-Cresol, .alpha.,.alpha.'-(tetram 265622 104978-24-7 38
                 ethylenedinitrilo)bis[6-methoxy-
                 7-Chloro-2,3-dihydrofuro(2,3-b)qui  81289 064124-91-0 35
                 noline

 50  23.517  0.00 C:\MassHunter\LIBRARY\NIST20.L
```

Library Search Report

Data Path : C:\GCMS Data\05-06-2024\
Data File : S678.D
Acq On : 06 Jun 2024 19:14
Operator :
Sample : S-6, 7, 8
Misc :
ALS Vial : 7 Sample Multiplier: 1

Search Libraries: C:\MassHunter\LIBRARY\NIST20.L Minimum Quality: 0

Unknown Spectrum: Apex
Integration Events: ChemStation Integrator - autoint1.e

Pk#	RT	Area%	Library/ID	Ref#	CAS#	Qual
			3-Amino-4,6-dimethylthieno[2,3-b]pyridine-2-carbonitrile	78573	052505-57-4	50
			Silane, diethylethoxy(2-methylpent-3-yloxy)-	113554	1000363-77-1	47
			Acetamide, N-(3-methylphenyl)- 2,2,2-trifluoro-	79271	1000307-30-9	47
51	24.291	0.02	C:\MassHunter\LIBRARY\NIST20.L			
			Phytol	194551	000150-86-7	86
			Phytol	194548	000150-86-7	50
			4-Fluorobenzoic acid, 2-tetrahydrofurylmethyl ester	103841	1000279-07-0	47
52	24.736	0.00	C:\MassHunter\LIBRARY\NIST20.L			
			9,12-Octadecadienoic acid (Z,Z)-	173583	000060-33-3	91
			7-Pentadecyne	85355	022089-89-0	86
			1,8,11-Heptadecatriene, (Z,Z)-	116386	056134-03-3	50
53	24.868	0.02	C:\MassHunter\LIBRARY\NIST20.L			
			9,12,15-Octadecatrien-1-ol, (Z,Z,Z)-	153474	000506-44-5	43
			Docosapentaenoic Acid methyl ester	253739	108698-02-8	38
			6,4'-Dimethoxy-3-hydroxyflavone	196652	093176-02-4	30
54	25.507	0.00	C:\MassHunter\LIBRARY\NIST20.L			
			9-Cyano-7,8-dimethyl-2-oxo-1H-2,3,4,5-tetrahydropyrrolo[1,2-a]-1,3-diazepine	78609	060138-29-6	46
			1-Phenyl-3-methyl-4-oximido-2-pyrazolin-5-one	78551	001080-89-3	38
			Bis(3,3,5,5-tetramethylcyclohexyl) diethylpyrophosphonate (isomer 2)	333579	1000510-34-6	38
55	26.445	0.05	C:\MassHunter\LIBRARY\NIST20.L			
			Cannabidivarol	181829	024274-48-4	99
			p-Heptylacetophenone	96751	037593-03-6	64
			Eupatoriochromene	96442	019013-03-7	59
56	26.865	0.04	C:\MassHunter\LIBRARY\NIST20.L			
			2-Methyl-6,7-methylenedioxy-4[1H]quinolone	78653	1000227-48-2	64
			4-Hydrazino-6-pyridin-2-yl-[1,3,5]triazin-2-ylamine	79159	175204-69-0	64
			4H-Pyrazolo[3,4-b]pyrane-5-carbonitrile, 6-amino-4-phenyl-3-propyl-	173183	300393-93-5	50
57	27.160	0.01	C:\MassHunter\LIBRARY\NIST20.L			
			Phenanthrene, 1,2,3,4,4a,10a-hexahydro-7-methoxy-1,1,4a-trimethyl-8-(1-methylethyl)-	197473	054833-49-7	55
			3-(2,5-Dimethylthiophen-3-yl)-2-(4-methoxyphenyl)cyclopent-2-en-1-on	196958	1000444-65-9	38
			5-Isopentyl-6-methyl-2-(methylsulfanyl)-4-pyrimidinol, TMS derivativ	196253	1000297-98-3	38

Data Path : C:\GCMS Data\05-06-2024\
Data File : S678.D
Acq On : 06 Jun 2024 19:14
Operator :
Sample : S-6, 7, 8
Misc :
ALS Vial : 7 Sample Multiplier: 1

Search Libraries: C:\MassHunter\LIBRARY\NIST20.L Minimum Quality: 0

Unknown Spectrum: Apex
Integration Events: ChemStation Integrator - autoint1.e

Pk#	RT	Area%	Library/ID	Ref#	CAS#	Qual
58	27.388	0.00	C:\MassHunter\LIBRARY\NIST20.L			
			3-Chloro-8-methylthio-11H-indolo[3,2-c]quinoline	196451	155249-85-7	38
			1,2,3-Trimethoxy-5-[2-(4-methoxyphenyl)ethynyl]benzene	197046	208347-68-6	38
			Bisphenol C, acetate	197221	1000462-16-1	27
59	27.572	0.01	C:\MassHunter\LIBRARY\NIST20.L			
			1H-4-Oxabenzo(f)cyclobut(cd)inden-8-ol, 1a-.alpha.,2,3,3a,8b-.alpha.,8c-.alpha.-hexahydro-1,1,3a-trimethyl-6-pentyl-	217712	021366-63-2	87
			Cannabichromene	217656	020675-51-8	87
			Cannabidiol	217644	013956-29-1	83
60	27.847	0.02	C:\MassHunter\LIBRARY\NIST20.L			
			Dimethyl 2,4-quinolinedicarboxylat	129287	007170-24-3	30
			2-Amino-4-(4-chlorophenyl)-7-hydroxy-4H-chromene-3-carbonitrile	196449	1000468-82-0	30
			Pyrido[3,4-d]pyrimidin-4(3H)-one, 2,6,8-trimethyl-	63536	022378-52-5	27
61	28.061	0.34	C:\MassHunter\LIBRARY\NIST20.L			
			.delta.9-Tetrahydrocannabivarin	181849	031262-37-0	99
			9-Oxabicyclo[4.3.0]non-6-en-8-one, 7-[2-methylenebicyclo[3.3.0]octane-3,6-dione-1-yl]-	181622	1000160-28-9	60
			1-(2-Methoxyphenyl)-2,5-dihydro-1H-pyrrole-2,5-dione	78674	017392-68-6	59
62	28.260	0.00	C:\MassHunter\LIBRARY\NIST20.L			
			Indole, TMS derivative	63751	017983-42-5	43
			Benzeneethanol, .beta.-methoxy-.beta.-(trifluoromethyl)-, (S)-	98144	052356-17-9	43
			Thiazole, 4-methyl-2-(phenylmethyl)-	63740	007210-74-4	43
63	28.358	0.01	C:\MassHunter\LIBRARY\NIST20.L			
			1H-4-Oxabenzo(f)cyclobut(cd)inden-8-ol, 1a-.alpha.,2,3,3a,8b-.alpha.,8c-.alpha.-hexahydro-1,1,3a-trimethyl-6-pentyl-	217711	021366-63-2	70
			1H-4-Oxabenzo(f)cyclobut(cd)inden-8-ol, 1a-.alpha.,2,3,3a,8b-.alpha.,8c-.alpha.-hexahydro-1,1,3a-trimethyl-6-pentyl-	217712	021366-63-2	70
			Cannabidiol	217643	013956-29-1	62
64	28.479	0.01	C:\MassHunter\LIBRARY\NIST20.L			
			8-Acetyl-6-(4-nitrophenyl)-2,3-dihydroindolizin-5(1H)-one	196509	1000482-06-5	80
			2,4-Dimethyl-6-(1-phenylethyl)phenol, trimethylsilyl ether	197268	1000462-25-6	80

Library Search Report

```
Data Path : C:\GCMS Data\05-06-2024\
Data File : S678.D
Acq On    : 06 Jun 2024  19:14
Operator  :
Sample    : S-6, 7, 8
Misc      :
ALS Vial  : 7   Sample Multiplier: 1

Search Libraries:   C:\MassHunter\LIBRARY\NIST20.L    Minimum Quality:   0

Unknown Spectrum:   Apex
Integration Events: ChemStation Integrator - autoint1.e

Pk#     RT  Area%          Library/ID                Ref#     CAS#   Qual
___________________________________________________________________________
                8-Acetyl-6-(3-nitrophenyl)-2,3-dih 196508 1000482-06-4 64
                ydroindolizin-5(1H)-one

 65  28.644  0.02 C:\MassHunter\LIBRARY\NIST20.L
                Cannabispiran                      130823 061262-81-5 95
                6-(2-Methylbutyl)oxy-4-methylcouma 130830 1000395-87-1 64
                rin
                6-(Neopentyl)oxy-4-methylcoumarin  130829 1000395-92-4 64

 66  28.844  0.09 C:\MassHunter\LIBRARY\NIST20.L
                6H-Dibenzo[b,d]pyran-1-ol, 6,6,9-t 176320 033745-21-0 94
                rimethyl-3-propyl-
                1H-Trindene, 2,3,4,5,6,7,8,9-octah 176422 040650-56-4 72
                ydro-1,1,4,4,7,7-hexamethyl-
                6-Aminonicotinic acid,N,O di-TMS   175212 1000472-37-7 72

 67  29.141  0.00 C:\MassHunter\LIBRARY\NIST20.L
                2-Ethylhydroquinone, bis(trimethyl 175513 1000463-15-0 70
                silyl) ether
                9-Ethyl-N-(trimethylsilyl)-9H-carb 176078 1000473-81-1 70
                azol-3-amine
                9-Ethyl-N-(trimethylsilyl)-9H-carb 176077 1000473-81-1 70
                azol-3-amine

 68  29.256  0.00 C:\MassHunter\LIBRARY\NIST20.L
                2-(4-Methoxyphenyl)-6-methyl-1-(py 217620 1000435-25-2 52
                ridin-3-yl)indole
                Pyrene, 1,6-bis(1,1-dimethylethyl) 217751 055044-29-6 45
                2-Nitrofluorenone, 4-methylphenyli 217542 111796-34-0 43
                mine

 69  29.440  0.13 C:\MassHunter\LIBRARY\NIST20.L
                Cannabidiol                        217645 013956-29-1 99
                Cannabidiol                        217646 013956-29-1 98
                Cannabidiol                        217639 013956-29-1 98

 70  29.623  0.18 C:\MassHunter\LIBRARY\NIST20.L
                Cannabichromene                    217655 020675-51-8 97
                Cannabichromene                    217657 020675-51-8 96
                Cannabichromene                    217653 020675-51-8 95

 71  29.926  0.02 C:\MassHunter\LIBRARY\NIST20.L
                Pregn-17(20)-en-16-one, (5.alpha., 200066 054548-12-8 64
                17Z)-
                4a(2H)-Phenanthrenecarboxaldehyde, 217710 057397-33-8 60
                 1,3,4,9,10,10a-hexahydro-6-methox
                y-1,1-dimethyl-7-(1-methylethyl)-,
                 (4aR-trans)-
                Morphinan-6-one, 4,5-epoxy-2-hydro 180164 076786-92-0 46
                xy-N-methyl-, (5.alpha.)-

 72  30.171  0.01 C:\MassHunter\LIBRARY\NIST20.L
                exo-THC                            217632 027179-28-8 64
                .DELTA.8-Tetrahydrocannabinol      217676 005957-75-5 45
```

Data Path : C:\GCMS Data\05-06-2024\
Data File : S678.D
Acq On : 06 Jun 2024 19:14
Operator :
Sample : S-6, 7, 8
Misc :
ALS Vial : 7 Sample Multiplier: 1

Search Libraries: C:\MassHunter\LIBRARY\NIST20.L Minimum Quality: 0

Unknown Spectrum: Apex
Integration Events: ChemStation Integrator - autoint1.e

Pk#	RT	Area%	Library/ID	Ref#	CAS#	Qual
			Benz[b]-1,4-oxazepine-4(5H)-thione, 2,3-dihydro-2,8-dimethyl-	83671	1000258-63-4	44
73	30.366	0.00	C:\MassHunter\LIBRARY\NIST20.L			
			Cannabidiol	217640	013956-29-1	41
			Ethyl 5-(4-methylphenyl)-1,3-oxazole-2-carboxylate	112607	033115-91-2	41
			Cannabidiol	217641	013956-29-1	41
74	30.574	0.63	C:\MassHunter\LIBRARY\NIST20.L			
			Dronabinol	217638	001972-08-3	99
			Dronabinol	217637	001972-08-3	99
			Dronabinol	217635	001972-08-3	99
75	30.877	0.01	C:\MassHunter\LIBRARY\NIST20.L			
			4-((Bis(trimethylsilyl)amino)methyl)-N-(trimethylsilyl)benzenesulfonamide	303288	1000485-72-1	38
			Silane, diethylheptadecyloxy(2-methoxyethoxy)-	311456	1000363-54-6	38
			Skullcapflavone II, dimethyl ether	303499	074670-18-1	32
76	31.192	0.17	C:\MassHunter\LIBRARY\NIST20.L			
			Cannabinol	212244	000521-35-7	99
			Cannabinol	212246	000521-35-7	98
			Cannabinol	212247	000521-35-7	97
77	31.495	0.01	C:\MassHunter\LIBRARY\NIST20.L			
			3-Bromo-5,6,7,8-tetrahydro-1,6-naphthyridine, N-trifluoroacetyl-	208480	1000506-47-2	56
			7-Ethoxy-3-(4-methoxyphenyl)-4-methylcoumarin	211972	263364-88-1	52
			2-Propanone, 1-phenyl-1-(5-phenyl-3H-1,2-dithiol-3-ylidene)-	211733	038489-98-4	49
78	31.772	0.00	C:\MassHunter\LIBRARY\NIST20.L			
			Tetrasiloxane, decamethyl-	210888	000141-62-8	43
			N-(3-Phenylpropyl)pyridine-3-carboxamide, TMS derivative	214408	1000510-98-3	38
			N-(2-Hydroxyethyl)pyridine-3-carboxamide, 2TMS derivative	211233	1000485-24-9	38
79	31.968	0.01	C:\MassHunter\LIBRARY\NIST20.L			
			4-(2,4-Dichlorophenyl)-5-methyl-2-thiazolamine, TMS	235995	1000512-01-0	60
			4-Ethoxy-N-(3,4,5-trimethoxybenzylidene)aniline	218497	158319-78-9	25
			Benzo[f]quinolin-3(2H)-one, 1,4-dihydro-1-(4-isopropylphenyl)-	218758	1000272-51-8	25
80	32.076	0.01	C:\MassHunter\LIBRARY\NIST20.L			
			Anthracene, 1,2,3,4,5,6,7,8-octahydro-1,1,4,4,5,5,8,8-octamethyl-	197476	022306-30-5	38
			Spiro[2,5-cyclohexadiene-1,7'(1'H)	177365	002241-43-2	35

Library Search Report

Data Path : C:\GCMS Data\05-06-2024\
Data File : S678.D
Acq On : 06 Jun 2024 19:14
Operator :
Sample : S-6, 7, 8
Misc :
ALS Vial : 7 Sample Multiplier: 1

Search Libraries: C:\MassHunter\LIBRARY\NIST20.L Minimum Quality: 0

Unknown Spectrum: Apex
Integration Events: ChemStation Integrator - autoint1.e

```
Pk#     RT  Area%          Library/ID                 Ref#     CAS#   Qual
__________________________________________________________________________
                 -cyclopent[ij]isoquinolin]-4-one,
                 2',3',8',8'a-tetrahydro-5'-hydroxy
                 -6'-methoxy-, (R)-
                 Ethoxy(phenyl)silanediol, 2TMS     233553 1000496-86-0 25

 81  32.236  0.01 C:\MassHunter\LIBRARY\NIST20.L
                 6-Hydroxyflavone, trimethylsilyl e 211811 1000454-60-4 78
                 ther
                 7-Hydroxyflavone, trimethylsilyl e 211814 039273-05-7 78
                 ther
                 6-Hydroxyflavone, trimethylsilyl e 211806 1000454-60-4 78
                 ther

 82  32.337  0.01 C:\MassHunter\LIBRARY\NIST20.L
                 Eicosane                           176384 000112-95-8 97
                 Heneicosane                        194590 000629-94-7 93
                 Heptacosane                        287976 000593-49-7 91

 83  32.939  0.00 C:\MassHunter\LIBRARY\NIST20.L
                 2-Methyl-7-phenylindole             84013 001140-08-5 43
                 Arsenous acid, tris(trimethylsilyl 251605 055429-29-3 38
                 ) ester
                 1,2-Benzisothiazol-3-amine, TBDMS  152650 1000332-57-2 38
                 derivative

 84  33.214  0.00 C:\MassHunter\LIBRARY\NIST20.L
                 Tetrasiloxane, decamethyl-         210886 000141-62-8 47
                 Methyltris(trimethylsiloxy)silane  210890 017928-28-8 47
                 2-Methyl-7-phenylindole             84013 001140-08-5 46

 85  33.724  0.01 C:\MassHunter\LIBRARY\NIST20.L
                 2-Propanesulfonamide, N-[2-(4'-cya 250811 211311-95-4 45
                 no[1,1'-biphenyl]-4-yl)propyl]-
                 9,10-Methanoanthracen-11-ol, 9,10- 136133 126615-74-5 41
                 dihydro-9,10,11-trimethyl-
                 2-Bromo-4,5-dimethoxycinnamic acid 180502 051314-72-8 38

 86  33.830  0.00 C:\MassHunter\LIBRARY\NIST20.L
                 (E)-3-(3,4-Dimethoxyphenyl)prop-2-  83611 130973-10-3 35
                 enamide
                 1,1,1,3,5,5,5-Heptamethyltrisiloxa 102314 001873-88-7 35
                 ne
                 Indolizine, 2-(4-methylphenyl)-     84034 007496-81-3 30

 87  33.924  0.03 C:\MassHunter\LIBRARY\NIST20.L
                 Eicosane                           176384 000112-95-8 97
                 Octadecane                         141057 000593-45-3 97
                 Pentacosane                        262013 000629-99-2 93

 88  34.890  0.02 C:\MassHunter\LIBRARY\NIST20.L
                 Cyclotrisiloxane, hexamethyl-      102257 000541-05-9 46
                 Methyltris(trimethylsiloxy)silane  210890 017928-28-8 43
                 Cyclotrisiloxane, hexamethyl-      102259 000541-05-9 43
```

Library Search Report

```
Data Path : C:\GCMS Data\05-06-2024\
Data File : S678.D
Acq On    : 06 Jun 2024  19:14
Operator  :
Sample    : S-6, 7, 8
Misc      :
ALS Vial  : 7   Sample Multiplier: 1

Search Libraries:   C:\MassHunter\LIBRARY\NIST20.L    Minimum Quality:   0

Unknown Spectrum:   Apex
Integration Events: ChemStation Integrator - autoint1.e

Pk#     RT  Area%          Library/ID                Ref#     CAS#   Qual
_____________________________________________________________________________
 89  35.351  0.03 C:\MassHunter\LIBRARY\NIST20.L
                 Arsenous acid, tris(trimethylsilyl 251605 055429-29-3 52
                 ) ester
                 Cyclotrisiloxane, hexamethyl-      102257 000541-05-9 50
                 Ethoxy(phenyl)silanediol, 2TMS     233553 1000496-86-0 50

 90  35.854  0.01 C:\MassHunter\LIBRARY\NIST20.L
                 Tetrasiloxane, decamethyl-         210888 000141-62-8 59
                 Cyclotrisiloxane, hexamethyl-      102257 000541-05-9 58
                 Arsenous acid, tris(trimethylsilyl 251605 055429-29-3 58
                 ) ester

 91  36.045  0.00 C:\MassHunter\LIBRARY\NIST20.L
                 Cyclotrisiloxane, hexamethyl-      102257 000541-05-9 58
                 1,1,1,3,5,5,5-Heptamethyltrisiloxa 102314 001873-88-7 50
                 ne
                 Arsenous acid, tris(trimethylsilyl 251605 055429-29-3 50
                 ) ester

 92  36.251  0.01 C:\MassHunter\LIBRARY\NIST20.L
                 Tetrasiloxane, decamethyl-         210888 000141-62-8 47
                 Cyclotrisiloxane, hexamethyl-      102257 000541-05-9 46
                 Arsenous acid, tris(trimethylsilyl 251605 055429-29-3 46
                 ) ester

 93  36.481  0.00 C:\MassHunter\LIBRARY\NIST20.L
                 Cyclotrisiloxane, hexamethyl-      102257 000541-05-9 52
                 Arsenous acid, tris(trimethylsilyl 251605 055429-29-3 52
                 ) ester
                 Tris(tert-butyldimethylsilyloxy)ar 330994 1000366-57-5 50
                 sane

 94  37.047  0.02 C:\MassHunter\LIBRARY\NIST20.L
                 .gamma.-Sitosterol                 310597 000083-47-6 91
                 .beta.-Sitosterol                  310596 000083-46-5 53
                 .gamma.-Sitosterol                 310598 000083-47-6 53

 95  37.446  0.01 C:\MassHunter\LIBRARY\NIST20.L
                 2-Methyl-7-phenylindole             84013 001140-08-5 46
                 Benzo[h]quinoline, 2,4-dimethyl-    84038 000605-67-4 43
                 Tris(tert-butyldimethylsilyloxy)ar 330994 1000366-57-5 43
                 sane

 96  37.944  0.01 C:\MassHunter\LIBRARY\NIST20.L
                 5-Ethyl 11a-methyl (5S,11aS)-6,11- 248337 1000484-03-0 56
                 dihydro-3H-indolizino[6,7-b]indole
                 -5,11a(5H)-dicarboxylate
                 1-(3,5-Di-tert-butyl-2,6-dihydroxy 153209 1000443-79-6 47
                 phenyl)ethanone
                 4-tert-Octylphenol, TMS derivative 171116 078721-87-6 43

 97  38.406  0.01 C:\MassHunter\LIBRARY\NIST20.L
                 Tris(tert-butyldimethylsilyloxy)ar 330994 1000366-57-5 46
                 sane
```

Library Search Report

```
Data Path : C:\GCMS Data\05-06-2024\
Data File : S678.D
Acq On    : 06 Jun 2024  19:14
Operator  :
Sample    : S-6, 7, 8
Misc      :
ALS Vial  : 7   Sample Multiplier: 1

Search Libraries:   C:\MassHunter\LIBRARY\NIST20.L    Minimum Quality:   0

Unknown Spectrum:   Apex
Integration Events: ChemStation Integrator - autoint1.e
```

Pk#	RT	Area%	Library/ID	Ref#	CAS#	Qual
			Tetrasiloxane, decamethyl-	210888	000141-62-8	43
			Octylsilanetriol, 3TMS	306483	1000496-84-7	43
98	38.695	0.01	C:\MassHunter\LIBRARY\NIST20.L			
			Arsenous acid, tris(trimethylsilyl) ester	251605	055429-29-3	49
			4-Methyl-2-trimethylsilyloxy-aceto phenone	101293	097389-70-3	49
			1H-Indole, 1-methyl-2-phenyl-	84028	003558-24-5	47
99	38.949	0.01	C:\MassHunter\LIBRARY\NIST20.L			
			Tris(tert-butyldimethylsilyloxy)ar sane	330994	1000366-57-5	53
			Arsenous acid, tris(trimethylsilyl) ester	251605	055429-29-3	52
			Cyclotrisiloxane, hexamethyl-	102259	000541-05-9	50
100	39.659	0.00	C:\MassHunter\LIBRARY\NIST20.L			
			Tetrasiloxane, decamethyl-	210888	000141-62-8	50
			Arsenous acid, tris(trimethylsilyl) ester	251605	055429-29-3	49
			Cyclotrisiloxane, hexamethyl-	102257	000541-05-9	49
